# Research Progress on Sesquiterpenoids of *Curcumae Rhizoma* and Their Pharmacological Effects

**DOI:** 10.3390/biom14040387

**Published:** 2024-03-23

**Authors:** Ting Cui, Bo-Yu Li, Fei Liu, Liang Xiong

**Affiliations:** 1 State Key Laboratory of Southwestern Chinese Medicine Resources, School of Pharmacy, Chengdu University of Traditional Chinese Medicine, Chengdu 611137, China; cuiting@stu.cdutcm.edu.cn (T.C.); boyuli@stu.cdutcm.edu.cn (B.-Y.L.); 2 Institute of Innovative Medicine Ingredients of Southwest Specialty Medicinal Materials, Chengdu University of Traditional Chinese Medicine, Chengdu 611137, China; 3 School of Medical Technology, Chengdu University of Traditional Chinese Medicine, Chengdu 611137, China

**Keywords:** *Curcumae Rhizoma*, sesquiterpenoids, chemical constituents, pharmacological activity

## Abstract

*Curcumae Rhizoma*, a traditional Chinese medicine with a wide range of pharmacological activities, is obtained from the dried rhizomes of *Curcuma phaeocaulis* VaL., *Curcuma kwangsiensis* S. G. Lee et C. F. Liang, and *Curcuma wenyujin* Y. H. Chen et C. Ling. Sesquiterpenoids and curcuminoids are found to be the main constituents of *Curcumae Rhizoma*. Sesquiterpenoids are composed of three isoprene units and are susceptible to complex transformations, such as cyclization, rearrangement, and oxidation. They are the most structurally diverse class of plant-based natural products with a wide range of biological activities and are widely found in nature. In recent years, scholars have conducted abundant studies on the structures and pharmacological properties of components of *Curcumae Rhizoma*. This article elucidates the chemical structures, medicinal properties, and biological properties of the sesquiterpenoids (a total of 274 compounds) isolated from *Curcumae Rhizoma*. We summarized extraction and isolation methods for sesquiterpenoids, established a chemical component library of sesquiterpenoids in *Curcumae Rhizoma*, and analyzed structural variances among sesquiterpenoids sourced from *Curcumae Rhizoma* of diverse botanical origins. Furthermore, our investigation reveals a diverse array of sesquiterpenoid types, encompassing guaiane-type, germacrane-type, eudesmane-type, elemane-type, cadinane-type, carane-type, bisabolane-type, humulane-type, and other types, emphasizing the relationship between structural diversity and activity. We hope to provide a valuable reference for further research and exploitation and pave the way for the development of new drugs derived from medicinal plants.

## 1. Introduction

Natural products encompass secondary metabolites crafted by organisms over millions of years of natural evolution, showcasing a plethora of diverse chemical structures. Human life is intricately connected to natural products, serving as a primary source of numerous medicinal drugs or pivotal lead compounds. Sesquiterpenoids are a class of natural products consisting of three isoprene units with structurally diverse basic skeletons. They are derived from farnesyl pyrophosphate (FPP), formed from three molecules of isopentenyl pyrophosphate (IPP), through a series of complex transformations, including cyclization, rearrangement, and oxidation. Although the basic skeleton of sesquiterpenoids contains only 15 carbons, the number of sesquiterpenoids is the highest among terpenoids. Emerging evidence has shown that these compounds have multifaceted biological activities, including, but not limited to, anti-inflammatory, cytotoxic, antitumor, hepatoprotective, and cardiovascular disease-improving properties, both in vitro in cell models and in vivo in animal models [1,2,3,4].

*Curcumae Rhizoma* (Ezhu) is the dried rhizomes of *Curcuma phaeocaulis* VaL., *Curcuma kwangsiensis* S. G. Lee et C. F. Liang, and *Curcuma wenyujin* Y. H. Chen et C. Ling [5]. It is an important traditional Chinese medicine commonly used in clinical practice for treating dysmenorrhea, amenorrhea, irregular menstruation, stasis in the pelvis, tumors of the abdomen and epigastrium, arrhythmia, coronary heart disease, stroke, dyspepsia, and gastritis [6]. In the modern world, *Curcumae Rhizoma* attracts great interest because of its various pharmacological effects on gynecological-related, cancer-related, immune system-related, cardiovascular system-related, and hepatoprotective activities, which mainly overlap with its traditional applications [7,8,9,10,11,12,13,14]. The major bioactive compounds of *Curcumae Rhizoma* are sesquiterpenoids and curcuminoids [15].

To date, numerous experimental studies have been conducted on the sesquiterpenoids in *Curcumae Rhizoma* [2,16,17,18]. However, there are fewer reviews on the sesquiterpenoids and their bioactivities in Ezhu. Some reviews primarily focus on a specific activity, including its effects on cancer, hepatobiliary disease, and infectious diseases [9,10,19,20,21], while others concentrate on the differences between several herbs derived from the genus *Curcuma* (*Curcumae Longae Rhizoma*, *Curcumae Radix*, and *Curcumae Rhizoma*) [6,7]. Accordingly, in this article, we review the sesquiterpenoids derived from the dried rhizomes of *C. phaeocaulis*, *C. kwangsiensis*, and *C. wenyujin*, three sources of *Curcumae Rhizoma*, and emphasize the structural variances among sesquiterpenoids sourced from diverse botanical origins. Additionally, we also summarize the structural features of different types of sesquiterpenoids and their pharmacological activities, revealing the relationship between structural diversity and activity. These discussions aim to serve as a reference and provide foundational knowledge for the prospective advancement and exploitation of *Curcumae Rhizoma*.

## 2. Medicinal Plants of *Curcumae Rhizoma*

Ezhu, a traditional Chinese medicine, comes from the genus *Curcuma* in the family Zingiberaceae. There are approximately 80 species of the genus *Curcuma* worldwide, mainly produced in Southeast Asia and from southeastern to southwestern regions in China [7]. *Curcuma phaeocaulis*, *Curcuma kwangsiensis*, *Curcuma longa*, *Curcuma zanthorrhiza*, *Curcuma wenyujin*, *Curcuma aeruginosa*, *Curcuma zedoaria*, and *Curcuma caesia* all belong to this genus [22]. The rhizomes are usually the main commercial sources of *Curcumae Rhizoma*, *Curcumae Longae Rhizoma*, or *Wenyujin Rhizoma Concisum*, while the tuberous roots are the main source of *Curcumae Radix* [7]. However, complicated relationships exist between these herbs, and there is confusion with respect to their application due to the similarity of their efficacy, the intersection of and variation in plant sources, and the overlap of herb and plant names. In addition, some plant sources are not included in the Pharmacopoeia of the people’s Republic of China, although they are widely used in folklore medicine. According to the Chinese Pharmacopoeia, *Curcumae Rhizoma* (Ezhu) only comes from the dried rhizomes of *C. phaeocaulis*, *C. kwangsiensis*, and *C. wenyujin*.

## 3. Chemical Composition of *Curcumae Rhizoma*

Through modern research, it has been discovered that volatile oil and curcuminoids are the main bioactive constituents of *Curcumae Rhizoma*, and the volatile oil predominantly comprises sesquiterpenoids [15]. These sesquiterpenoids are of various types, including guaiane-type, germacrane-type, eudesmane-type, elemane-type, cadinane-type, carane-type, bisabolane-type, humulane-type, and other types.

A wide range of published studies have revealed the isolation and identification of sesquiterpenoids with diverse structural skeletons. Considering operability in the laboratory, sesquiterpenoids are mainly obtained by organic solvent extraction and steam distillation. Nevertheless, since conventional extraction techniques have several drawbacks, such as long times of extraction or the use of large amounts of solvents, the use of green extraction techniques is suggested, without affecting the efficiency of the extraction. When employing steam distillation, some thermally unstable components are prone to degradation. Chemical compounds are purified primarily by repeated column chromatography, including silica gel column chromatography, reversed-phase C18 silica gel column chromatography, Sephadex LH-20 column chromatography, ODS column chromatography, HPLC, and preparative TLC. Compared to other compounds, sesquiterpenoids possess lower polarity, as well as differences in their affinity and solubility in organic phases. When using silica gel column chromatography, several solvent systems are generally used for elution, including petroleum ether–acetone, petroleum ether–EtOAc, CHCl_3_–MeOH, CH_2_Cl_2_–MeOH, CH_2_Cl_2_–EtOAc, and CH_2_Cl_2_–acetone. The structures of isolated compounds were established based on 1D and 2D NMR data, mass spectrometry, circular dichroism (CD), X-ray analysis, and chemical methods. In comparison with other types of sesquiterpenoids, guaiane-type, germacrane-type, and eudesmane-type sesquiterpenoids are prone to recrystallization, suggesting that recrystallization may be a consideration when the configuration is undetermined.

### 3.1. Guaiane-Type Sesquiterpenoids of Curcumae Rhizoma

**Figure 1:**
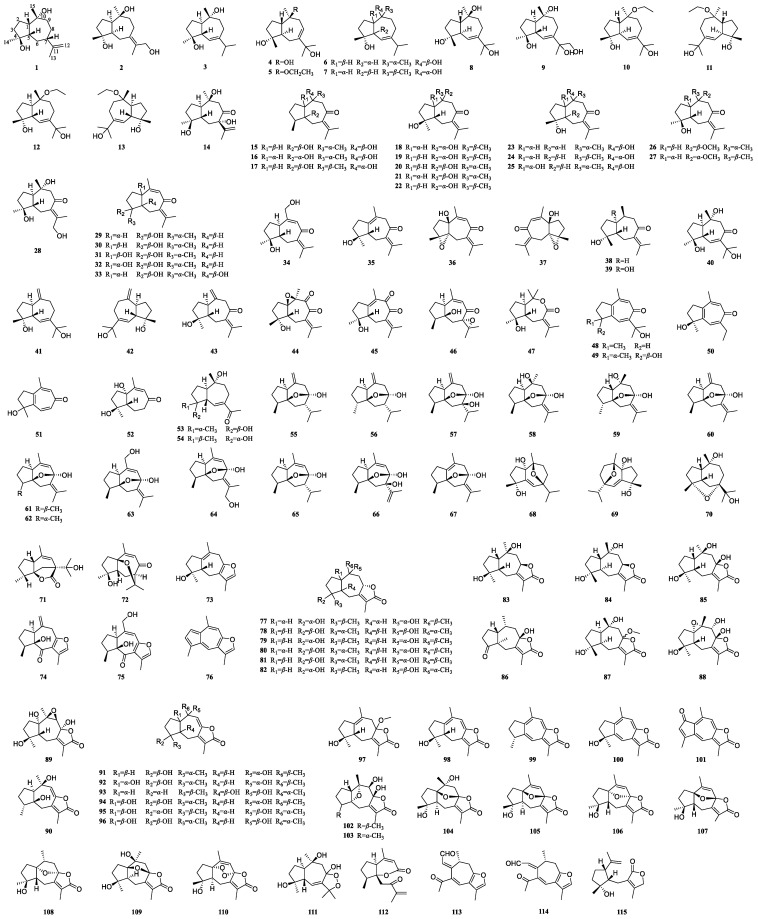
Guaiane-type sesquiterpenoids of *Curcumae Rhizoma*.

Guaiane-type sesquiterpenoids are the most dominant type of sesquiterpenoids in *Curcumae Rhizoma*. They are characterized by a five-membered ring fused to a seven-membered ring. To date, 115 guaiane-type sesquiterpenoids have been isolated from three medicinal sources (*C. phaeocaulis*, *C. kwangsiensis*, and *C. wenyujin*) (Figure 1, Table 1). Roughly one-fourth of these compounds form a five-membered lactone ring between C-8 and C-12 (**77**–**110**, **115**), and six additional compounds produce a furan ring at the C-8 and C-12 positions (**73**–**76**, **113**, **114**). This type of compounds tend to generate oxygen bridges at various positions, including C-5/C-8 (**55**–**67**), C-7/C-10 (**68**, **69**), C-5/C-10 (**102**, **103**), and C-1/C-8 (**104**–**109**); in addition, compound **110** features a peroxide bridge between C-1 and C-8. Distinctively, several seco-guaiane-type sesquiterpenoids exist, and compounds **112** and **115** are subjected to ring opening on the seven-membered ring, while compounds **113** and **114** are opened at C-3–C-4. It is noteworthy that these guaiane-type sesquiterpenoids tend to possess hydroxyl groups at C-4, C-5, C-8, and C-10. Moreover, they are readily oxidized to carbonyl groups at C-8 and easily generate double bonds and oxygen rings, which make them structurally diverse. These compounds feature multiple chiral carbons, which lead to various stereoisomers, diastereoisomers, enantiomers (**10/11**, **12/13**, **36/37**, **41/42**, **68/69**, **77/83**), and epimers (**29/30**, **31/32**, **61/62**), among which all enantiomers originate from *C. phaeocaulis*.

In a comparative analysis of the distribution of sesquiterpenoids across three plant sources, guaiane-type sesquiterpenoids exhibited a predominant presence in *C. wenyujin* and *C. phaeocaulis*, with a marked pre-eminence in abundance noted specifically in *C. wenyujin*. Conversely, bicyclic sesquiterpenoids demonstrated a significant association with *C. wenyujin* and *C. phaeocaulis*, while sesquiterpenoids with a furan ring or a lactone ring were mainly found in *C. wenyujin* and *C. kwangsiensis*. In addition, while each of the three botanical specimens shares certain chemical constituents, their contents exhibit notable divergence. Specifically, *C. kwangsiensis* demonstrates a notable enrichment in curcumol (**55**), while *C. phaeocaulis* showcases the highest proportions of isocurcumenol (**60**) and curcumenol (**61**) among the three plants. These findings highlight the nuanced variations in compound distribution across closely related plant species [23,24,25,26].

**Table 1:** Guaiane-type sesquiterpenoids of *Curcumae Rhizoma*.

No.	Compounds	Medicinal Source	Reference
**1**	*epi*-Guaidiol A	*C. wenyujin*	[27]
**2**	Phaeocaulisguatriol	*C. phaeocaulis*	[28]
**3**	Alismoxide	*C. wenyujin*, *C. phaeocaulis*, *C. kwangsiensis*	[12,16,29,30]
**4**	4*α*,10*α*,11-Trihydroxy-1*β*H,5*β*H-guai-7(8)-ene	*C. phaeocaulis*	[28]
**5**	Wenyujinol E	*C. wenyujin*	[27]
**6**	Guaianediol	*C. phaeocaulis*, *C. wenyujin*	[27,28]
**7**	6-Guaiene-4*α*,10*α*-diol	*C. wenyujin*	[31]
**8**	4*α*,10*β*,11-Trihydroxy-1,5-trans-guai-6-ene	*C. phaeocaulis*	[28]
**9**	Wenyujinol N	*C. wenyujin*	[32]
**10**	(+)-Phaeocauline A	*C. phaeocaulis*	[33]
**11**	(−)-Phaeocauline A	*C. phaeocaulis*	[33]
**12**	(+)-Phaeocauline B	*C. phaeocaulis*	[33]
**13**	(−)-Phaeocauline B	*C. phaeocaulis*	[33]
**14**	Phaeocaulisin Q	*C. phaeocaulis*	[34]
**15**	Wenyujinin A	*C. wenyujin*, *C. kwangsiensis*	[12,35]
**16**	Wenyujinin B	*C. wenyujin*	[27,35]
**17**	Wenyujinin Q	*C. wenyujin*	[36]
**18**	Zedoarondiol	*C. wenyujin*, *C. phaeocaulis*, *C. kwangsiensis*	[16,27,31,37,38]
**19**	Neozedoarondiol	*C. wenyujin*	[10]
**20**	Isozedoarondiol	*C. wenyujin*, *C. phaeocaulis*, *C. kwangsiensis*	[16,27,28,36,38]
**21**	Phaeocaulisin E	*C. wenyujin*, *C. phaeocaulis*	[16,31,37]
**22**	(1*S*,4*S*,5*S*,10*R*)-Zedoarondiol	*C. phaeocaulis*, *C. wenyujin*, *C. kwangsiensis*	[16,38,39]
**23**	(1*S*,4*S*,5*S*,10*R*)-Isozedoarondiol	*C. wenyujin*	[31]
**24**	Wenyujinin R	*C. wenyujin*	[36]
**25**	4,10-Epizedoarondiol	*C. kwangsiensis*, *C. wenyujin*	[31,38]
**26**	4-Hydroxy-10-methoxy-guai-7(11)-en-8-one	*C. phaeocaulis*	[28]
**27**	Methylzedoarondiol	*C. wenyujin*	[27]
**28**	Wenyujinol M	*C. wenyujin*	[32]
**29**	Procurcumenol	*C. wenyujin*, *C. phaeocaulis*, *C. kwangsiensis*	[12,27,29,31,37]
**30**	Epiprocurcumenol	*C. wenyujin*	[40]
**31**	Aerugidiol	*C. wenyujin*, *C. kwangsiensis*	[29,38,39]
**32**	1-*epi*-Aerugidiol	*C. phaeocaulis*	[37]
**33**	Procurcumadiol	*C. wenyujin*, *C. phaeocaulis*, *C. kwangsiensis*	[16,31,37,38]
**34**	Phaeocaulisin F	*C. phaeocaulis*	[16]
**35**	Neoprocurcumenol	*C. wenyujin*	[36]
**36**	(+)-Phaeocauline D	*C. phaeocaulis*	[33]
**37**	(−)-Phaeocauline D	*C. phaeocaulis*	[33]
**38**	Dihydroprocurcumenol	*C. kwangsiensis*	[12]
**39**	Wenyujinol D	*C. wenyujin*	[27]
**40**	Phaeocaulisin P	*C. phaeocaulis*	[34]
**41**	(+)-Phaeocauline E	*C. phaeocaulis*	[33]
**42**	(−)-Phaeocauline E	*C. phaeocaulis*	[33]
**43**	Isoprocurcumenol	*C. wenyujin*	[41]
**44**	Wenyujinol F	*C. wenyujin*	[27]
**45**	9-Oxo-neoprocurcumenol	*C. wenyujin*	[27]
**46**	7*α*,11*α*-Epoxy-5*β*-hydroxy-9-guaiane-8-one	*C. wenyujin*, *C. phaeocaulis*	[16,31]
**47**	8,9-seco-4*β*-Hydroxy-1*α*,5*β*H-7(11)-guaen-8,10-olide	*C. wenyujin*	[29]
**48**	Phaeocaulisin L	*C. phaeocaulis*	[42]
**49**	Phaeocaulisin D	*C. phaeocaulis*	[16]
**50**	Phaeocaulisin R	*C. phaeocaulis*	[37]
**51**	Phaeocaulisin K	*C. phaeocaulis*	[42]
**52**	Phaeocaulisin J	*C. phaeocaulis*	[16,28]
**53**	4*α*,10*β*-Dihydroxy-1*β*H,5*α*H-guai-6(7)-en-11-one	*C. phaeocaulis*	[34]
**54**	Phaeocaulisin N	*C. phaeocaulis*	[34]
**55**	Curcumol	*C. wenyujin*, *C. kwangsiensis*, *C. phaeocaulis*	[26,31,36,43]
**56**	4-Epicurcumol	*C. wenyujin*	[44]
**57**	7*β*,8*α*-Dihydroxy-1*α*,4*α*H-guai-10(15)-en-5*β*,8*β*-endoxide	*C. wenyujin*	[29]
**58**	10*β*-Hydroxy-9,10-dihydrocurcumenol	*C. phaeocaulis*	[28]
**59**	Wenyujinin I	*C. wenyujin*, *C. kwangsiensis*	[12,35]
**60**	Isocurcumenol	*C. phaeocaulis*, *C. wenyujin*, *C. kwangsiensis*	[16,26,45]
**61**	Curcumenol	*C. wenyujin*, *C. phaeocaulis*, *C. kwangsiensis*	[12,16,28,31,46]
**62**	4-Epicurcumenol	*C. wenyujin*, *C. phaeocaulis*	[16,46]
**63**	15-Hydroxycurcumenol	*C. phaeocaulis*	[28]
**64**	12-Hydroxycurcumenol	*C. wenyujin*	[31]
**65**	Isocurcumol	*C. wenyujin*	[44]
**66**	7*β*,8*α*-dihydroxy-1*α*,4*α*H-guai-9,11-dien-5*β*,8*β*-endoxide	*C. wenyujin*	[46]
**67**	Neocurcumenol	*C. wenyujin*	[46]
**68**	(+)-Phaeocauline C	*C. phaeocaulis*	[33]
**69**	(−)-Phaeocauline C	*C. phaeocaulis*	[33]
**70**	4*α*,7*α*-Epoxyguaiane-10*α*,11-diol	*C. wenyujin*	[32]
**71**	(1*R*,4*R*,5*S*,7*S*)-Curwenyujinone	*C. wenyujin*	[47]
**72**	Wenyujinin H	*C. wenyujin*	[35]
**73**	Curcumafuranol	*C. kwangsiensis*	[48]
**74**	Zedoarol	*C. kwangsiensis*	[49]
**75**	Wenyujinin F	*C. wenyujin*	[35]
**76**	Linderazulene	*C. kwangsiensis*	[50]
**77**	(+)-Zedoalactone A	*C. wenyujin*, *C. phaeocaulis*	[28,51]
**78**	Zedoalactone C	*C. wenyujin*, *C. phaeocaulis*, *C. kwangsiensis*	[16,52,53]
**79**	Zedoalactone E	*C. wenyujin*	[39,46]
**80**	Zedoalactone G	*C. wenyujin*, *C. kwangsiensis*	[51,52]
**81**	Zedoalactone H	*C. wenyujin*	[46]
**82**	Phaeocaulisin C	*C. phaeocaulis*, *C. kwangsiensis*	[16,52]
**83**	Zedoalactone A	*C. wenyujin*, *C. kwangsiensis*, *C. phaeocaulis*	[16,28,31,52]
**84**	Phaeocaulisin B	*C. phaeocaulis*	[16]
**85**	Zedoarolide B	*C. wenyujin*, *C. phaeocaulis*, *C. kwangsiensis*	[12,16,28,31,51]
**86**	Wenyujinol H	*C. wenyujin*	[27]
**87**	8-*O*-Methylzedoarolide B	*C. wenyujin*	[32]
**88**	Zedoarolide A	*C. phaeocaulis*, *C. wenyujin*	[16,28,32]
**89**	Wenyujinol G	*C. wenyujin*	[27]
**90**	Phaeocaulisin I	*C. phaeocaulis*, *C. kwangsiensis*	[12,16]
**91**	Phaeocaulisin G	*C. phaeocaulis*	[16]
**92**	Phaeocaulisin H	*C. phaeocaulis*	[16]
**93**	Phaeocaulisin O	*C. kwangsiensis*, *C. phaeocaulis*	[34,52]
**94**	Zedoalactone B	*C. wenyujin*, *C. kwangsiensis*, *C. phaeocaulis*	[16,27,51,52]
**95**	(1*R*,4*R*,5*S*,10*S*)-Zedoalactone B	*C. wenyujin*	[51]
**96**	Zedoalactone D	*C. wenyujin*, *C. kwangsiensis*, *C. phaeocaulis*	[16,39,52]
**97**	(4*S*)-Hydroxy-(8)-methoxy-(5*S*)-(H)-guaia1(10),7(11)-dien-12,8-olide	*C. kwangsiensis*	[12]
**98**	Zedoalactone F	*C. wenyujin*, *C. kwangsiensis*	[38,39]
**99**	Gweicurculactone	*C. kwangsiensis*	[54]
**100**	(4*S*)-4-Hydroxy-gweicurculactone	*C. wenyujin*, *C. kwangsiensis*	[51,54]
**101**	2-Oxoguaia-1(10),3,5,7(11),8-pentaen-12,8-olide	*C. wenyujin*, *C. kwangsiensis*	[27,54]
**102**	4*β*-Methyl-8*β*,9*β*-dihydroxy-5*α*,10*α*-epoxy-guai-12,8-olide	*C. kwangsiensis*	[52]
**103**	4*α*-Methyl-8*β*,9*β*-dihydroxy-5*α*,10*α*-epoxy-guai-12,8-olide	*C. kwangsiensis*	[52]
**104**	Phaeocaulisin A	*C. phaeocaulis*, *C. kwangsiensis*	[16,54]
**105**	(1*R*,4*R*,5*S*,8*S*,9*Z*)-4-Hydroxy-1,8-epoxy-5H-guaia-7(11),9-dien-12,8-olide	*C. kwangsiensis*	[54]
**106**	Wenyujinol A	*C. wenyujin*	[27]
**107**	Wenyujinol B	*C. wenyujin*	[27]
**108**	Wenyujinol C	*C. wenyujin*	[27]
**109**	Wenyujinin G	*C. wenyujin*	[35]
**110**	1*α*,8*α*-Epidioxy-4*α*-hydroxy-5*α*H-guai-7(11),9-dien-12,8-olide	*C. wenyujin*, *C. kwangsiensis*	[12,29]
**111**	Phaeocaulisin M	*C. phaeocaulis*	[42]
**112**	Curcuzedoalide	*C. wenyujin*	[31]
**113**	Kwangsiensis A	*C. kwangsiensis*	[55]
**114**	Kwangsiensis B	*C. kwangsiensis*	[55]
**115**	12-Dehydroxy-chloraniolide	*C. phaeocaulis*	[56]

### 3.2. Germacrane-Type Sesquiterpenoids of Curcumae Rhizoma

**Figure 2:**
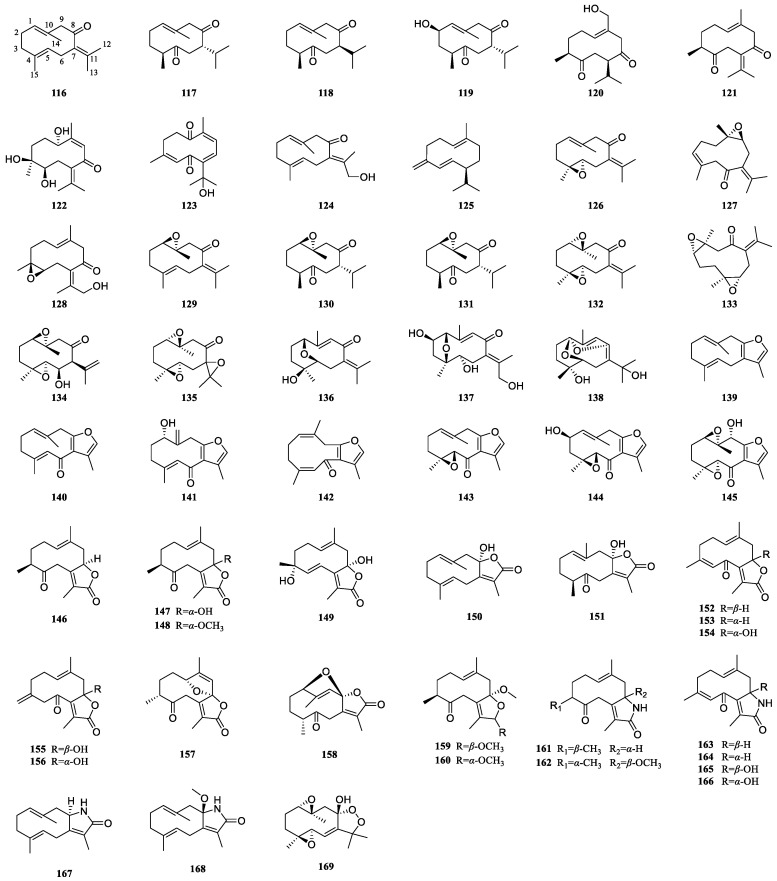
Germacrane-type sesquiterpenoids of *Curcumae Rhizoma*.

Germacrane-type sesquiterpenoids, a notably abundant class among numerous sesquiterpenoids, can generate distinct sesquiterpenoids, including guaiane-, eudesmane-, and cadinane-type sesquiterpenoids. Members of this class of compounds typically contain one or more double bonds, which are formed at C-1/C-10 and C-4/C-5. These sesquiterpenoids can be separated into four different configurations due to the cis-trans isomerism of the double bonds. The readily deformable 10-membered rings inherent in germacrane sesquiterpenoids result in a diverse array of stereo structures. Currently, 54 germacrane sesquiterpenoids have been isolated from *Curcumae Rhizoma* (Figure 2, Table 2). These natural products frequently engage in the formation of a five-membered ring at C-8 and C-12, yielding diverse structural moieties, such as furan rings (**139**–**145**), lactone rings (**146**–**158**), and lactam rings (**161**–**168**). This type of compound is prone to oxidation, which can produce aldehydes, ketones, esters, or oxygen bridges. Specifically, the C-5, C-7, and C-8 positions are particularly susceptible to oxidation, resulting in carbonyl groups. Germacrane-type sesquiterpenoids containing a lactone ring are frequently substituted with hydroxyl groups at C-8 (**147**, **149**–**151**, **154**–**156**). Sesquiterpenoids are also prone to forming oxygen bridges, with tricyclic oxygen rings appearing frequently at C-1/C-10 (**129**–**135**), C-4/C-5 (**126**, **128**, **143**–**145**), or C-1/C-5 (**136**). It is noteworthy that multiple pairs of germacrane-type enantiomers (**152**/**153**, **155**/**156**, **163**/**164**, **165**/**166**) have been isolated from *Curcumae Rhizoma*. All these enantiomers originate from *C. phaeocaulis*, implying the pervasive presence of germacrane-type enantiomers in *C. phaeocaulis*.

Germacrane-type sesquiterpenoids are obtained from *C. wenyujin* and *C. phaeocaulis*, with a relatively low occurrence in *C. kwangsiensis*. Notably, compounds characterized by oxygen bridges exhibit an almost exclusive presence within *C. wenyujin*, while sesquiterpenoids featuring either a furan or a lactone ring manifest a consistent and uniform distribution across three plant species. The studies elucidate a commonality in the presence of certain compounds across all three plants. Among them, germacrone (**116**), curdione (**117**), neocurdione (**118**), germacrene D (**125**), and furanodiene (**139**) exhibit higher concentrations in *C. wenyujin* compared to the other two botanical specimens. Conversely, furanodienone (**140**) attains greater levels in *C. phaeocaulis*. Notably, dehydrocurdione (**121**) levels in *C. kwangsiensis* are relatively high. These findings underscore the nuanced variations in the phytochemical compositions among closely related plant species, accentuating the unique metabolic pathways shaping the distinctive chemical profiles of *C. wenyujin*, *C. kwangsiensis*, and *C. phaeocaulis* [23,24,25,26].

**Table 2:** Germacrane-type sesquiterpenoids of *Curcumae Rhizoma*.

No.	Compounds	Medicinal Source	Reference
**116**	Germacrone	*C. wenyujin*, *C. kwangsiensis*, *C. phaeocaulis*	[31,49,57]
**117**	Curdione	*C. wenyujin*, *C. kwangsiensis*, *C. phaeocaulis*	[17,26,29,31,43]
**118**	Neocurdione	*C. wenyujin*, *C. kwangsiensis*, *C. phaeocaulis*	[26,43,44,58]
**119**	(2R)-2β-Hydroxycurdione	*C. wenyujin*	[18]
**120**	Wenyujinone D	*C. wenyujin*	[18]
**121**	Dehydrocurdione	*C. kwangsiensis*	[12]
**122**	Heyneanone C	*C. phaeocaulis*	[59]
**123**	Heyneanone D	*C. wenyujin*	[18,40]
**124**	13-Hydroxygermacrone	*C. wenyujin*, *C. phaeocaulis*, *C. kwangsiensis*	[31,56,60]
**125**	Germacrene D	*C. wenyujin*, *C. phaeocaulis*, *C. kwangsiensis*	[26]
**126**	(4S,5S)-Germacrone-4,5-epoxide	*C. wenyujin*, *C. kwangsiensis*, *C. phaeocaulis*	[49,57,58,61]
**127**	(+)-(4S,5S)-Germacrone-4,5-epoxide	*C. wenyujin*	[17,62]
**128**	(4S,5S)-13-Hydroxygermacrone-4,5-epoxide	*C. phaeocaulis*	[59]
**129**	Germacrone-1,10-epoxide	*C. wenyujin*, *C. kwangsiensis*	[49,58]
**130**	(1R,10R)-(−)-1,10-Dihydrocurdione	*C. wenyujin*	[63]
**131**	(1R,10R)-Epoxy-1,10-dihydrocurdione	*C. wenyujin*	[43]
**132**	(1S,10S),(4S,5S)-Germacrone-1(10),4(5)-diepoxide	*C. wenyujin*	[43,62]
**133**	(+)-(1S,4S,5S,10S)-Germacrone-1(10)-4-diepoxide	*C. wenyujin*	[17]
**134**	(1R,4S,5R,6R,7S,10R)-1(10),4(5)-Diepoxygermacran-11(12)-en-6-ol	*C. phaeocaulis*	[15]
**135**	Germacrone-1(10),4,7(11)-triepoxide	*C. wenyujin*	[62]
**136**	Wenyujinin J	*C. wenyujin*	[35]
**137**	Wenyujinol O	*C. wenyujin*	[32]
**138**	Phagermadiol	*C. phaeocaulis*	[42,59]
**139**	Furanodiene	*C. kwangsiensis*, *C. wenyujin*, *C. phaeocaulis*	[26]
**140**	Furanodienone	*C. wenyujin*, *C. kwangsiensis*, *C. phaeocaulis*	[31,49,56]
**141**	(1S)-1-Hydroxy-isofuranodienone	*C. phaeocaulis*	[37]
**142**	1(10)Z,4Z-Furanodiene-6-one	*C. wenyujin*	[31]
**143**	Zederone	*C. wenyujin*, *C. kwangsiensis*, *C. phaeocaulis*	[31,49,56]
**144**	Wenyujinin K	*C. wenyujin*	[35]
**145**	(1R,4S,5R,9R,10S)-9-Hydroxy-zederone epoxide	*C. phaeocaulis*	[59]
**146**	Curdionolide B	*C. wenyujin*, *C. phaeocaulis*, *C. kwangsiensis*	[12,17,44,59]
**147**	Curdionolide A	*C. wenyujin*, *C. phaeocaulis*, *C. kwangsiensis*	[17,31,52,59]
**148**	Souliene A	*C. kwangsiensis*	[12]
**149**	Wenyujinone C	*C. wenyujin*	[18]
**150**	Aeruginolactone	*C. wenyujin*, *C. phaeocaulis*, *C. kwangsiensis*	[12,30,56]
**151**	Curcuminol G	*C. wenyujin*, *C. kwangsiensis*	[12,45]
**152**	(+)-Phaeocaulin C	*C. phaeocaulis*	[64]
**153**	(−)-Phaeocaulin C	*C. phaeocaulis*	[64]
**154**	(1E,4Z)-8-Hydroxy-6-oxogermacra-1(10),4,7(11)-trieno-12,8-lactone	*C. wenyujin*, *C. phaeocaulis*	[17,30,56]
**155**	(+)-Phaeocaulin D	*C. phaeocaulis*	[64]
**156**	(−)-Phaeocaulin D	*C. phaeocaulis*	[64]
**157**	Wenyujinone A	*C. wenyujin*	[18]
**158**	1,8-Epoxy-7(11)-germacren-5-one-12,8-olide	*C. wenyujin*	[18]
**159**	Curkwangsien A	*C. kwangsiensis*	[65]
**160**	Curkwangsien B	*C. kwangsiensis*	[65]
**161**	Curdionolide C	*C. wenyujin*	[17]
**162**	Wenyujinone B	*C. wenyujin*	[18]
**163**	(+)-Phaeocaulin B	*C. phaeocaulis*	[64]
**164**	(−)-Phaeocaulin B	*C. phaeocaulis*	[64]
**165**	(+)-Phaeocaulin A	*C. phaeocaulis*	[59]
**166**	(−)-Phaeocaulin A	*C. phaeocaulis*	[59]
**167**	(−)-Phaeocaulin E	*C. phaeocaulis*	[56]
**168**	(+)-Phaeocaulin F	*C. phaeocaulis*	[56]
**169**	Wenjine	*C. wenyujin*	[62]

### 3.3. Eudesmane-Type Sesquiterpenoids of Curcumae Rhizoma

**Figure 3:**
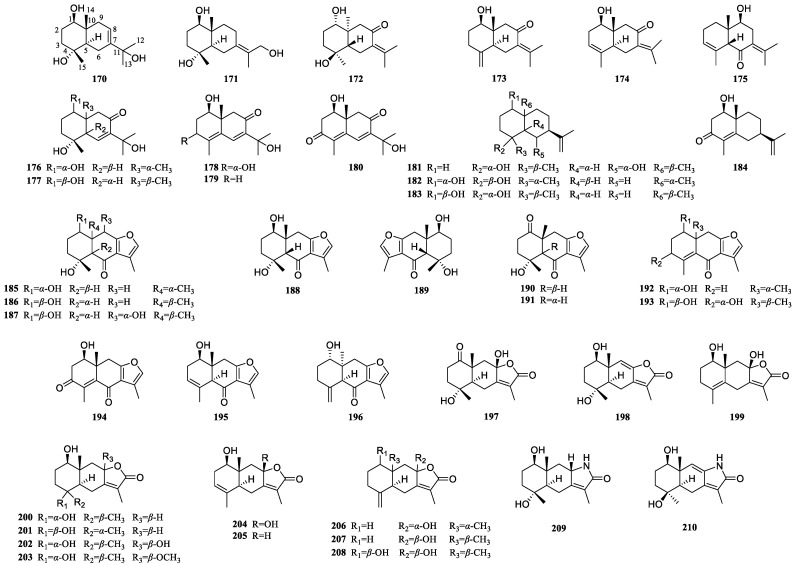
Eudesmane-type sesquiterpenoids of *Curcumae Rhizoma*.

Eudesmane-type sesquiterpenoids are a common type of natural product, whose fundamental structure comprises two six-membered rings. Previous studies have identified 41 eudesmane sesquiterpenoids from *Curcumae Rhizoma* (Figure 3, Table 3). These natural products are likely to form furan rings (**185**–**196**), lactone rings (**197**–**208**), and lactam rings (**209** and **210**) at the C-8 and C-12 positions. They are highly prone to oxidation and dehydrogenation, resulting in hydroxyl and carbonyl groups and double bonds. Among these, hydroxyl substitutions often occur at the C-1, C-4, and C-11 positions, and carbonyl substitution occurs at the C-6 and C-8 positions. Some compounds are oxidized to carbonyl groups at C-1 and C-4, while those at C-3/C-4, C-4/C-5, C-7/C-8, C-8/C-9, C-7/C-11, C-11/C-12, and C-4/C-15 positions are often dehydrogenated to form double bonds. Eudesmane-type sesquiterpenoids typically possess three or more chiral carbons, resulting in a diverse range of conformations, among which a pair of enantiomers has been identified (**188** and **189**). Of these isolated compounds, the majority originated from *C. phaeocaulis*, with only three compounds from *C. kwangsiensis.*

### 3.4. Elemane-Type Sesquiterpenoids of Curcumae Rhizoma

**Figure 4:**
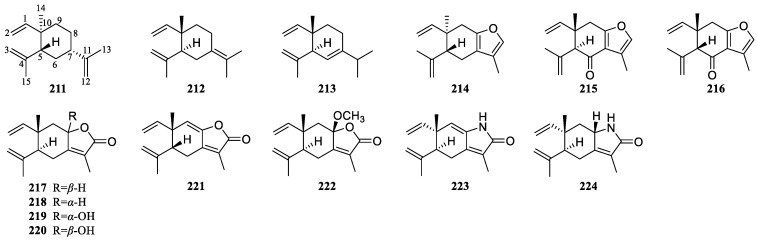
Elemane-type sesquiterpenoids of *Curcumae Rhizoma*.

At present, 14 elemane-type sesquiterpenoids have been reported from *Curcumae Rhizoma* (Figure 4, Table 4). Of these, some are monocyclic elemane-type sesquiterpenoids (**211**–**213**), and others form furan rings (**214**–**216**), five-membered lactone rings (**217**–**222**), or five-membered lactam rings (**223** and **224**) between C-8 and C-12. This class of compounds contains multiple double bonds, which often present at the C-1/C-2 or C-3/C-4 positions, while some compounds also exhibit these at C-6/C-7, C-7/C-8, C-7/C-11, C-8/C-9, or C-11/C-12.

### 3.5. Cadinane-Type Sesquiterpenoids of Curcumae Rhizoma

**Figure 5:**
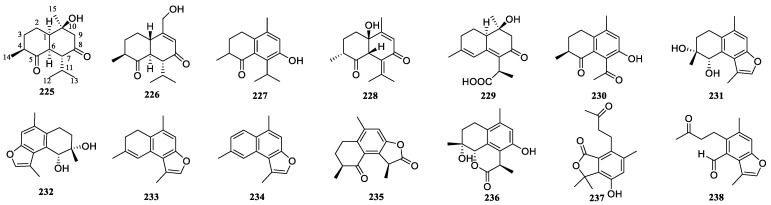
Cadinane-type sesquiterpenoids of *Curcumae Rhizoma*.

In total, 14 cadinane-type sesquiterpenoids have been identified from *Curcumae Rhizoma* (Table 5, Figure 5). Within this group, compounds **231**–**235** and **238** exhibit a furan ring or a five-membered lactone ring at the C-8/C-12 positions, while compound **236** has a six-membered lactone ring at the C-5/C-12 positions. Compounds **237** and **238** are subject to A-ring opening, and in certain instances, the B-ring acquires a benzene ring structure (**227**, **230**–**238**). These compounds are susceptible to oxidation at the C-5 position, leading to the generation of hydroxyl (**231** and **232**) or carbonyl groups (**225**–**228**, **230**, **235**, **237**, **238**). Furthermore, dehydrogenation readily occurs at C-4/C-5, giving rise to the formation of double bonds (**229**, **233**, **234**). Enantiomers are also present within the group of cadinane sesquiterpenoids (**231** and **232**).

### 3.6. Other-Type Sesquiterpenoids of Curcumae Rhizoma

**Figure 6:**
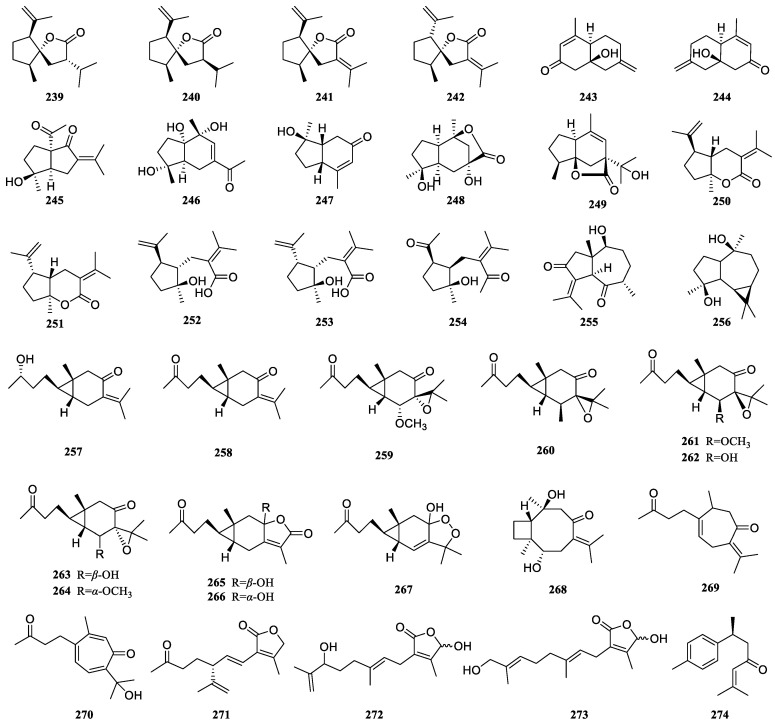
Other-type sesquiterpenoids of *Curcumae Rhizoma*.

Presently, 36 distinct sesquiterpenoids of *Curcumae Rhizoma* have been reported (Table 6, Figure 6), encompassing spironolactone-type sesquiterpenoids (**239**–**242**), carane-type sesquiterpenoids (**257**–**267**), bisabolane-type sesquiterpenoids (**274**), xanthane-type sesquiterpenoids (**269** and **270**), and diverse other-type sesquiterpenoids. These compounds typically show hydroxyl and carbonyl substitutions, among which compound **267** features a distinctive peroxy pentacyclic ring and compounds **259**–**264** each possess a three-membered oxygen ring. Intriguingly, compounds **239**/**240**, **241**/**242**, **250**/**251**, **252**/**253**, and **265**/**266** are epimers, while compounds **243**/**244** are enantiomers.

**Figure 7:**
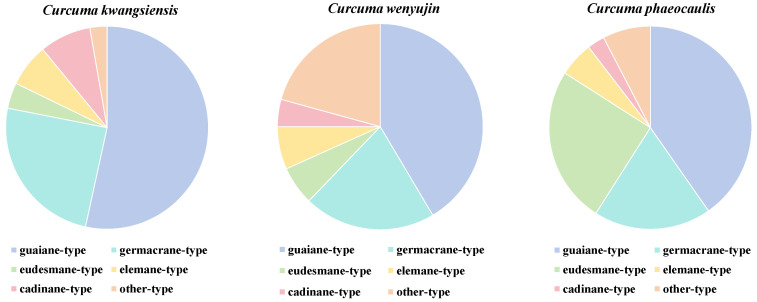
Distribution of sesquiterpenoids in three plant species.

**Figure 8:**
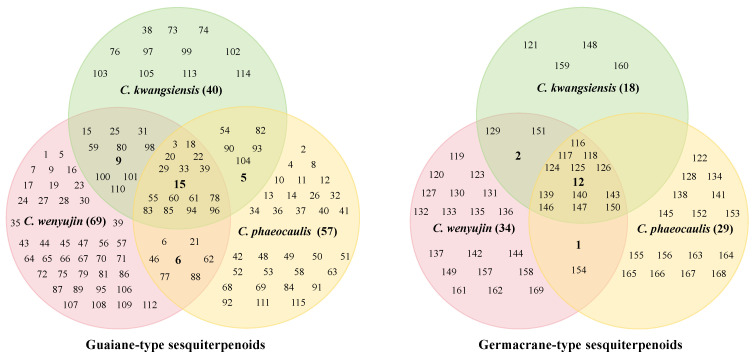
Distribution of guaiane-type and germacrane-type sesquiterpenoids in three medicinal herbs.

In summary, 74 sesquiterpenoids have been obtained from *C. kwangsiensis*, 160 from *C. wenyujin*, and 145 from *C. phaeocaulis.* As depicted in Figure 7, sesquiterpenoids originating from three distinct plants predominantly comprise guaiane- and germacrane-type sesquiterpenoids. Nevertheless, notable variations are also evident. For instance, eudesmane-type sesquiterpenoids are mainly found in *C. phaeocaulis*, while there is a higher abundance of sesquiterpenoids in *C. wenyujin*. Additionally, the proportion of guaiane-type sesquiterpenoids exhibit a markedly elevated level compared to other types of sesquiterpenoids in *C. kwangsiensis* (Figure 7). In a further analysis of the distribution patterns of guaiane- and germacrane-type sesquiterpenoids across the three medicinal plants, it can be observed that certain sesquiterpenoids are documented in two or three plants (Figure 8). The guaiane-type sesquiterpenoids shared among all three herbs include alismoxide (**3**), zedoarondiol (**18**), isozedoarondiol (**20**), (1*S*,4*S*,5*S*,10*R*)-zedoarondiol (**22**), procurcumenol (**29**), procurcumadiol (**33**), curcumol (**55**), isocurcumenol (**60**), curcumenol (**61**), zedoalactone C (**78**), zedoalactone A (**83**), zedoarolide B (**85**), zedoalactone B (**94**), and zedoalactone D (**96**); the germacrane-type sesquiterpenoids include germacrone (**116**), curdione (**117**), neocurdione (**118**), 13-hydroxygermacrone (**124**), germacrene D (**125**), (4*S*,5*S*)-germacrone-4,5-epoxide (**126**), furanodiene (**139**), furanodienone (**140**), zederone (**143**), curdionolide B (**146**), curdionolide A (**147**), and aeruginolactone (**150**). From the abovementioned results, it becomes apparent that the three medicinal plants exhibit similarities in sesquiterpenoids, featuring overlap. Notably, certain monomers are present in larger quantities in *Curcumae Rhizoma*, such as zedoarondiol (**18**), isozedoarondiol (**20**), procurcumenol (**29**), procurcumadiol (**33**), curcumol (**55**), isocurcumenol (**60**), curcumenol (**61**), germacrone (**116**), curdione (**117**), neocurdione (**118**), 13-hydroxygermacrone (**124**), furanodiene (**139**), and furanodienone (**140**), which indicates that all three sources can be utilized as substitutes for *Curcumae Rhizoma*, notwithstanding their diverse botanical origins. Additionally, in terms of the abundance of compounds, investigations into *C. wenyujin* exhibit greater depth, whereas research on *C. kwangsiensis* is relatively limited.

## 4. Biological Activity

It has been demonstrated that sesquiterpenoids in *Curcumae Rhizoma* have a wide range of pharmacological activities, including anti-inflammatory, cytotoxic, antitumor, anti-platelet aggregation, anti-atherosclerotic, hypoglycemic, hepatoprotective, antibacterial, anti-viral, antioxidant, anti-aging, neuroprotective, and anti-sepsis effects, as well as protective effects against myocardial ischemia–reperfusion injury.

### 4.1. Anti-Inflammatory Activity

Research has demonstrated that sesquiterpenoids in *Curcumae Rhizoma* exhibit remarkable anti-inflammatory activity (Table 7). Currently, research on anti-inflammatory activity primarily employs three different models: the lipopolysaccharide (LPS)-induced RAW 246.7 cell inflammation model, the LPS-induced THP-1 cell inflammation model, and the neuro-inflammatory model of LPS-stimulated BV-2 cells [75,76,77]. Further studies have revealed that some compounds, such as isozedoarondiol (**20**), phaeocaulisin D (**49**), curcumenol (**61**), 15-hydroxycurcumenol (**63**), zedoalactone B (**94**), phaeocaulisin M (**111**), phaeusmane B (**177**), curcolide (**202**), and curzerenone (**215**) have significant anti-inflammatory activities, with IC_50_ values ranging from 0.8 to 9.6 μM [2,16,42,56,78]. Subsequent mechanistic investigations revealed that sesquiterpenoids derived from *Curcumae Rhizoma* manifest their anti-inflammatory effects primarily by modulating the NF-*κ*B, MAPK, JAK2/STAT3, and ERK–MAPK signal pathways. In addition, some of the compounds can also play a role in other conditions triggered by inflammation, for example, by exerting anti-inflammatory and analgesic effects, ameliorating lung inflammation and inducing airway remodeling, treating the inflammation in bronchial asthma and rheumatoid arthritis, and others [12,79,80,81,82,83,84].

Comparing the structural types of the various anti-inflammatory components mentioned above, it can be observed that all types of sesquiterpenoids in *Curcumae Rhizoma* have certain anti-inflammatory activities, especially guaiane-type and eudesmane-type sesquiterpenoids, and their anti-inflammatory activities bear certain structure–activity relationships. The study by Xia [44] demonstrated that guaiane-type sesquiterpenoids are more effective than other types of sesquiterpenoids. The inhibitory effects of curcumalactone (**239**) are stronger than those of 7-epicurcumalactone (**240**), possibly because of the isopropyl group’s different spatial position at C-7 [68]. The anti-inflammatory activity of phaeocaulisin D (**49**) is stronger than that of phaeocaulisin L (**48**), and it is speculated that the hydroxyl group at C-4 can enhance the activity [85].

**Table 7:** Anti-inflammatory activity of sesquiterpenoids in *Curcumae Rhizoma*.

Compounds	Compound Types	Activity Types	Pharmacological Models	Effects	IC_50_ (μM)	Positive Control IC_50_ (μM)	Reference
Isozedoarondiol (**20**)	Guaiane-type sesquiterpenoids	Anti-inflammatory activity	LPS-induced RAW 246.7 cell inflammation model	Inhibit LPS-induced NO production	1.4	12.1 (Indomethacin) 43.8 (Hydrocortisone)	[16]
Phaeocaulisin L (**48**)					54.27 ± 4.23	58.66 ± 6.39 (Hydrocortisone)	[42]
Phaeocaulisin D (**49**)					5.9	12.1 (Indomethacin) 43.8 (Hydrocortisone)	[16]
Phaeocaulisin N (**54**)					3.58 ± 0.17	58.79 ± 3.32 (Hydrocortisone)	[34]
4-Epicurcumol (**56**)					17.26 ± 1.26	64.34 ± 7.49 (Hydrocortisone)	[44]
15-Hydroxycurcumenol (**63**)					6.44 ± 0.51	14.1 ± 0.69 (Indomethacin)	[78]
12-Hydroxycurcumenol (**64**)					9.64 ± 0.47	14.1 ± 0.69 (Indomethacin)	[78]
Isocurcumol (**65**)					22.36 ± 1.32	64.34 ± 7.49 (Hydrocortisone)	[44]
Zedoalactone A (**83**)					1.6	12.1 (Indomethacin) 43.8 (Hydrocortisone)	[16]
Phaeocaulisin B (**84**)					1.9	12.1 (Indomethacin) 43.8 (Hydrocortisone)	[16]
Zedoalactone B (**94**)					1.3	12.1 (Indomethacin) 43.8 (Hydrocortisone)	[16]
Zedoalactone D (**96**)					1.6	12.1 (Indomethacin) 43.8 (Hydrocortisone)	[16]
Phaeocaulisin A (**104**)					8.5	51.4 (Hydrocortisone)	[35]
Wenyujinin G (**109**)					7.6	51.4 (Hydrocortisone)	[35]
Phaeocaulisin M (**111**)					6.05 ± 0.43	58.66 ± 6.39 (Hydrocortisone)	[42]
Gweicurculactone (**99**)				Inhibit NO production and the expressions of iNOS and COX-2 mRNA	27.3	5.6 ± 0.3 (CAPE) 26.3 ± 0.3 (Indomethacin) 65.0 ± 1.2 (L-NA)	[86]
Curcuzedoalide (**112**)				Inhibit NO production and suppress pre-inflammatory protein expressions of iNOS and COX-2	12.21 ± 1.67	4.15 ± 1.35 (Quercetin)	[87]
4*α*,10*α*,11-Trihydroxy-1*β*H,5*β*H-guai-7(8)-ene (**4**)			LPS-induced THP-1 cell inflammation model	Inhibit the release of inflammatory mediator (TNF-α)			[88]
Zedoarondiol (**18**)			LPS-induced RAW 264.7 cell and mouse peritoneal macrophage cell models	Inhibit iNOS, COX-2, and pro-inflammatory cytokine (TNF-*α*, IL-1*β*, and IL-6) expressions by suppressing the phosphorylations of IKK and MAPKs, and inactivating the NF-*κ*B pathway			[89]
			LPS-induced THP-1-blue cell inflammation model	Inhibit LPS-stimulated TLR4 activation	22.5 ± 1.0	2.6 ± 0.8 (Luteolin)	[37]
Procurcumenol (**29**)		Anti neuro-inflammatory activity	LPS-induced BV-2 cell inflammation model	Inhibit LPS-induced NO production	20.05	23.53 ± 4.70 (Minocycline)	[52]
Dihydroprocurcumenol (**38**)		Anti-inflammatory activity	LPS-induced RAW 246.7 cell inflammation model	Inhibit the secretion of inflammatory mediator (COX-2)			[12]
		Anti-inflammatory and antinociceptive effects	Carrageenan-induced paw edema and acetic acid-induced writhing animal models	Inhibit the paw edema (inhibitory effects: 28.1% and 35.3% at 100 and 50 mg/kg, respectively); decrease the levels of stretching and twisting by the rates of 46.9%			[12]
Curcumol (**55**)		Anti-inflammatory activity	LPS-induced RAW 246.7 cell inflammation model	Suppress iNOS mRNA expression and protein level; inhibit the transcriptional and translational levels of TNF-*α*, IL-1*β*, and IL-6; interfere with the JNK-mediated AP-1 pathway			[90]
		Alleviate psoriasis-like inflammation activity	NHEK cell model	Reduce proliferation and inflammatory gene expression in stimulated keratinocytes by inhibiting JAK1/STAT3 signaling			[83]
		Ameliorate lung Inflammation activity	Asthmatic mice model established by ovalbumin induction	Inhibit the abnormal activation of the Wnt/β-catenin pathway			[82]
Curcumenol (**61**)		Anti-inflammatory activity	LPS-induced RAW 246.7 cell inflammation model	Inhibit the secretion of inflammatory mediators (COX-2, IL-1*β*, and TNF-*α*)			[12]
			LPS-induced macrophage inflammation model	Inhibit LPS-induced NO production	5.42 ± 0.64	14.1 ± 0.69 (Indomethacin)	[78]
		Anti neuro-inflammatory activity	LPS-induced BV-2 cell inflammation model	Inhibit releases of the inflammatory mediators (COX-2, IL-1*β*, and TNF-*α*) and diminish the expression of the regulatory genes by inhibiting Akt-dependent NF-*κ*B activation and downregulating Akt and p38 MAPK signaling			[91]
		Anti-inflammatory and antinociceptive effects	Carrageenan-induced paw edema and acetic acid-induced writhing animal models	Inhibit the paw edema (inhibitory effects: 29.5% and 30% at 100 and 50 mg/kg, respectively); decrease the levels of stretching and twisting by the rate of 32.7%			[12]
Neocurdione (**118**)	Germacrane-type sesquiterpenoids	Anti-inflammatory activity	LPS-induced RAW 246.7 cell inflammation model	Inhibit LPS-induced NO production	24.18 ± 1.66	64.34 ± 7.49 (Hydrocortisone)	[44]
Curdionolide B (**146**)					14.50 ± 0.87	64.34 ± 7.49 (Hydrocortisone)	[44]
Germacrone (**116**)		Anti-inflammatory activity; alleviate bronchial asthma and rheumatoid arthritis activity, etc.	Multiple inflammation models	Regulate the expressions of related genes and proteins by PI3K III/Beclin-1/Bcl-2 and PI3K/Akt/mTOR pathways; regulate the expression of pro-inflammatory cytokines (IL-6, TNF-*α*, TGF-*β*1, and IL-10); regulate Th1/Th2 balance and NF-*κ*B activation; upregulate TLR8 expression in THP-1 cells, etc.			[80]
		Alleviate rheumatoid arthritis activity	Collagen-induced arthritis (CIA) model	Alleviate the progression of arthritis through regulating Th1/Th2 balance and inactivating the NF-*κ*B pathway			[84]
Dehydrocurdione (**121**)		Analgesic activity; antipyretic activity; anti-inflammatory activity	Acetic acid-induced writhing method; baker’s yeast-treated rat model; carrageenan-induced paw edema model	Mitigate the writhing reflex induced by acetic acid and the fever elicited by baker’s yeast; inhibit the carrageenan-induced paw edema; reduce chronic adjuvant arthritis			[79]
Souliene A (**148**)		Anti-inflammatory activity	LPS-induced RAW 246.7 cell inflammation model	Inhibit the secretion of inflammatory mediator (COX-2)			[12]
		Anti-inflammatory and antinociceptive effects	Carrageenan-induced paw edema and acetic acid-induced writhing animal models	Inhibit the paw edema (inhibitory effects: 40.7% and 35.9% at 100 and 50 mg/kg, respectively); decrease the levels of stretching and twisting by the rate of 38.5%			[12]
Curcuminol G (**151**)		Anti-inflammatory activity	LPS-induced RAW 246.7 cell inflammation model	Inhibit the secretion of inflammatory mediators (COX-2, IL-1*β*, and TNF-*α*)			[12]
		Anti-inflammatory and antinociceptive effects	Carrageenan-induced paw edema and acetic acid-induced writhing animal models	Inhibit the paw edema (inhibitory effects: 31.4% and 45.4% at 100 and 50 mg/kg, respectively); decrease the levels of stretching and twisting by the rate of 26.2%			[12]
1*α*,4*β*-Dihydroxy-eudesm-7(11)-en-8-one (**172**)	Eudesmane-type sesquiterpenoids	Anti-inflammatory activity	LPS-induced RAW 246.7 cell inflammation model	Inhibit LPS-induced NO production	5.6	12.1 (Indomethacin) 43.8 (Hydrocortisone)	[2]
1-Hydroxyeudesma-4(14),7(11)-dien-8-one (**173**)					1.2	12.1 (Indomethacin) 43.8 (Hydrocortisone)	[2]
Phaeusmane A (**176**)					3.2	12.1 (Indomethacin) 43.8 (Hydrocortisone)	[2]
Phaeusmane B (**177**)					9.6	12.1 (Indomethacin) 43.8 (Hydrocortisone)	[2]
Phaeusmane D (**178**)					14.4	12.1 (Indomethacin) 43.8 (Hydrocortisone)	[2]
Phaeusmane C (**180**)					19.6	12.1 (Indomethacin) 43.8 (Hydrocortisone)	[2]
Eudesm-11-ene-4*α*,6*α*-diol (**181**)					0.8	12.1 (Indomethacin) 43.8 (Hydrocortisone)	[2]
1*β*-Hydroxyeudesma-4,11-dien-3-one (**184**)					9.3	12.1 (Indomethacin) 43.8 (Hydrocortisone)	[2]
Curcolonol (**186**)					16.2	12.1 (Indomethacin) 43.8 (Hydrocortisone)	[2]
Chlomultin B (**195**)					18.6	12.1 (Indomethacin) 43.8 (Hydrocortisone)	[2]
Myrrhterpenoid N (**196**)					19.3	12.1 (Indomethacin) 43.8 (Hydrocortisone)	[2]
Phaeusmane F (**197**)					4.8	12.1 (Indomethacin) 43.8 (Hydrocortisone)	[2]
(7*Z*)-1*β*,4*α*-Dihydroxy-5*α*,8*β*(H)-eudesm-7(11)-en-8,12-olide (**200**)					15.3	53.8 (Hydrocortisone)	[66]
(7*Z*)-1*β*,4*β*-Dihydroxy-5*α*,8*β*(H)-eudesm-7(11)-en-8,12-olide (**201**)					3.8	12.1 (Indomethacin) 43.8 (Hydrocortisone)	[2]
Curcolide (**202**)					0.8	12.1 (Indomethacin) 43.8 (Hydrocortisone)	[2]
1*β*,8*β*-Dihydroxy-eudesma-3,7(11)-dien-8*α*,12-olide (**204**)					8.9	12.1 (Indomethacin) 43.8 (Hydrocortisone)	[2]
Phaeusmane H (**210**)					20.9	12.1 (Indomethacin) 43.8 (Hydrocortisone)	[2]
Hydroxyisogermafurenolide (**220**)					26.0	53.8 (Hydrocortisone)	[66]
8*β*(H)-Elema-1,3,7(11),8-tetraen-8,12-lactam (**223**)					9.4 ± 1.6	42.7 ± 3.1 (Hydrocortisone)	[46]
Curzerenone (**215**)			IL-6-stimulated STAT-3 expression model	Inhibit STAT-3 expression stimulated by IL-6; suppress the mRNA expression levels of the proinflammatory genes IL-1*β* and CRP via blockade of the IL-6-activated and ERK-MAPK signaling pathways	4.8		[56]
Phacadinane B (**228**)	Cadinane-type sesquiterpenoids	Anti-inflammatory activity	LPS-induced RAW 246.7 cell inflammation model	Inhibit LPS-induced NO production	2.25 ± 0.71	43.80 ± 6.79 (Hydrocortisone)	[74]
Phacadinane A (**229**)					3.88 ± 0.58	43.80 ± 6.79 (Hydrocortisone)	[74]
Curcumalactone (**239**)	Other-type sesquiterpenoids	Anti-inflammatory activity	LPS-induced RAW 246.7 inflammation model	Inhibit LPS-induced NO production	23.28 ± 1.47	64.34 ± 7.49 (Hydrocortisone)	[68]
7-Epicurcumalactone (**240**)					45.49 ± 2.96	64.34 ± 7.49 (Hydrocortisone)	[68]
Phaeocauone (**246**)					2.35 ± 0.17	58.79 ± 3.32 (Hydrocortisone)	[69]
Phasalvione (**255**)					7.46 ± 0.69	58.79 ± 3.32 (Hydrocortisone)	[69]
8,11-Epidioxy-8-hydroxy-4-oxo-6-carabren (**267**)					25.36 ± 3.26	64.34 ± 7.49 (Hydrocortisone)	[44]
Curcumolide (**249**)				Suppress LPS-induced NF-*κ*B activation, including the nuclear translocation and DNA binding activity of NF-*κ*B; decrease pro-inflammatory mediators (TNF-*α*, IL-6, and IL-1*β*); NO and ROS production			[92]

### 4.2. Cancer-Related Activity

Sesquiterpenoids derived from *Curcumae Rhizoma* exhibit noteworthy efficacy against diverse tumor cell lines (Table 8), including ovarian, breast, cervical, gastric, leukemia, and various other malignancies. Ongoing research is particularly focused on exploring their impacts on breast cancer and hepatic cancer. Research on breast cancer has primarily focused on MCF-7 and MDA-MB-231 cell models. Studies have revealed the notable efficacy of furanodiene (**139**) in the context of breast cancer. This compound can inhibit the proliferation of breast cancer cells in multiple ways, including regulating cyclin D1, CDK2, pRb, and Bcl-2 family proteins, activating caspases and PARP in a mitochondria-mediated pathway, inhibiting cancer cell growth via the Akt pathway and the AMPK pathway, and inducing apoptosis via metabolic regulation [93,94]. In addition, sesquiterpenoids in *Curcumae Rhizoma*, including zedoarondiol (**18**), furanodiene (**139**), and *δ*-elemene (**213**), have cytotoxic activity against leukemia cells [95,96,97]. Furthermore, certain compounds, including curcumol (**55**), germacrone (**116**), furanodiene (**139**), and *β*-elemene (**211**), have been found to exhibit broad-spectrum cancer-related activity through various pathways [80,98,99,100,101].

In conclusion, all types of sesquiterpenoids in *Curcumae Rhizoma* exhibit cancer-related activities, and the main active substances are guaiane-type, germacrane-type, and elemane-type sesquiterpenoids. These compounds are evenly distributed among the three plants, with most of them being common to two or three of them. Some of the shared compounds have a high content and broad-spectrum cancer-related activity, inducing apoptosis in many types of cancer cells, and it is presumed that these compounds are the important material basis for *Curcumae Rhizoma*.

**Table 8:** Cancer-related activity of sesquiterpenoids in *Curcumae Rhizoma*.

Compounds	Compound Types	Activity Types	Pharmacological Models	Effects	IC_50_	Positive Control IC_50_	Reference
Zedoarondiol (**18**)	Guaiane-type sesquiterpenoids	Cytotoxic activity against lung carcinoma	A-549 cell model	Exhibit cytotoxic activity	3.64 ± 0.66 μM	0.0831 ± 0.0091 μM (Doxorubicin)	[95]
		Cytotoxic activity against breast cancer	MCF-7 cell model		7.34 ± 0.94 μM	8.02 ± 1.13 μM (Doxorubicin)	[95]
		Cytotoxic activity against breast cancer	MDA-MB-231 cell model		7.51 ± 1.35 μM	6.93 ± 1.08 μM (Doxorubicin)	[95]
		Cytotoxic activity against leukemia	HL-60 cell model		7.35 ± 0.61 μM	0.0776 ± 0.0082 μM (Doxorubicin)	[95]
Isozedoarondiol (**20**)		Cytotoxic activity against lung carcinoma	A-549 cell model		4.21 ± 0.93 μM	0.0831 ± 0.0091 μM (Doxorubicin)	[95]
		Cytotoxic activity against breast cancer	MCF-7 cell model		9.19 ± 0.79 μM	8.02 ± 1.13 μM (Doxorubicin)	[95]
		Cytotoxic activity against breast cancer	MDA-MB-231 cell model		9.40 ± 1.21 μM	6.93 ± 1.08 μM (Doxorubicin)	[95]
Phaeocaulisin E (**21**)		Cytotoxic activity against lung carcinoma	A-549 cell model		4.79 ± 0.81 μM	0.0831 ± 0.0091 μM (Doxorubicin)	[95]
		Cytotoxic activity against breast cancer	MCF-7 cell model		9.85 ± 1.02 μM	8.02 ± 1.13 μM (Doxorubicin)	[95]
		Cytotoxic activity against breast cancer	MDA-MB-231 cell model		10.15 ± 1.43 μM	6.93 ± 1.08 μM (Doxorubicin)	[95]
Procurcumenol (**29**)		Cytotoxic activity against lung carcinoma	A-549 cell model		5.82 ± 0.91 μM	0.0831 ± 0.0091 μM (Doxorubicin)	[95]
Aerugidiol (**31**)		Cytotoxic activity against breast cancer	MCF-7 cell model		7.23 ± 1.01 μM	8.02 ± 1.13 μM (Doxorubicin)	[95]
		Cytotoxic activity against breast cancer	MDA-MB-231 cell model		7.40 ± 0.93 μM	6.93 ± 1.08 μM (Doxorubicin)	[95]
Isoprocurcumenol (**43**)		Cytotoxic activity against lung carcinoma	A-549 cell model		3.81 ± 0.65 μM	0.0831 ± 0.0091 μM (Doxorubicin)	[95]
		Cytotoxic activity against breast cancer	MCF-7 cell model		8.13 ± 0.93 μM	8.02 ± 1.13 μM (Doxorubicin)	[95]
		Cytotoxic activity against breast cancer	MDA-MB-231 cell model		8.34 ± 1.14 μM	6.93 ± 1.08 μM (Doxorubicin)	[95]
Phaeocaulisguatriol (**2**)		Cytotoxic activity against breast cancer	MCF-7 cell model	Induce cell apoptosis by activating the expressions of TP53 and caspase 3 proteins	40.73 ± 0.42 μM	9.86 ± 0.13 μM (Cisplatin)	[28]
Curcumol (**55**)		Cytotoxic activity against lung carcinoma, breast cancer, nasopharyngeal carcinoma, etc.; antitumor activity against lung cancer, nasopharyngeal carcinoma, colorectal cancer, etc.	Multi-models	Arrest the cell cycle at G_0_/G_1_ or G_2_/M phases; induce apoptosis in numerous cancer cells via targeting key signaling pathways, such as MAPK/ERK, PI3K/Akt, and NF-*κ*B; regulate various signaling cascades			[98]
		Cytotoxic activity against breast cancer; antitumor activity against breast cancer	MDA-MB-231 cell model; MDA-MB-231 cell xenograft model in nude mice	Trigger apoptosis of p53 mutant triple-negative human breast cancer cells via activation of p73 and PUMA			[102]
		Cytotoxic activity against hepatic cancer; antitumor activity against hepatic cancer	Hela, A549, HUVEC cell models; Hep3B cell xenograft model in murine	Inhibit the expression of PD-L1 through crosstalk between HIF-1*α* and p-STAT3 (T705) signaling pathways			[103]
		Cytotoxic activity against colorectal cancer; antitumor activity against colorectal cancer	LoVo and SW 480 cell models; LoVo cell xenograft model in nude mice	Inhibit growth and induce apoptosis via IGF-1R and p38 MAPK pathways			[104]
Curcumenol (**61**)		Cytotoxic activity against breast cancer	MCF-7 cell model	Induce apoptosis by inhibiting the proliferation of the cancer cell	9.3 ± 0.3 μg/mL	0.1 ± 0.0 μg/mL (Doxorubicin)	[105]
		Cytotoxic activity against lung carcinoma; antitumor activity against lung carcinoma	CCD19, BEAS-2B, H1299, H460, and HEK293T cell models and mice xenograft model	Induce cell death, suppress cell proliferation, and trigger ferroptosis in lung cancer cells via the lncRNA H19/miR-19b-3p/FTH1 axis			[106]
Curcuzedoalide (**112**)		Cytotoxic activity against gastric cancer	AGS cell model	Activate caspase-8, caspase-9, caspase-3, and PARP, inducing apoptosis			[107]
Germacrone (**116**)	Germacrane-type sesquiterpenoids	Cytotoxic activity against colorectal cancer, gastric cancer, breast cancer, cervical cancer, prostate cancer, etc.	Multi-cell models	Regulate the expressions of Akt/MDM2/p53, JAK2/STAT3, AMPK, and Akt/mTOR pathways and related proteins; inhibit the proliferation of cancer cells, promote the apoptosis of cancer cells, promote autophagy; reverse the resistance of drugs, enhance the antitumor activity of drugs, and reduce the toxicity of chemotherapeutic drugs			[80]
		Cytotoxic activity against gastric cancer	BGC823 cell model	Inhibit cell proliferation through the induction of G_2_/M-phase cell cycle arrest and promote cell apoptosis through modulations of cell cycle-associated protein expression and mitochondria-mediated apoptosis			[108]
		Cytotoxic activity against breast cancer	MCF-7 and MDA-MB-23 cell models	Induce cell cycle arrest and apoptosis through mitochondria-mediated caspase pathway			[109]
		Cytotoxic activity against hepatic carcinoma	HepG_2_ and Bel7402 cell models	Regulate the expression of proteins related to G_2_/M cell cycle and apoptosis; p53 and oxidative damage may be involved in the inhibition of human hepatoma cells’ growth			[3]
		Cytotoxic activity against esophageal squamous cell carcinoma	Esophageal squamous cell carcinoma (ESCC) cell models	Exert an anti-esophageal effect through intrinsic apoptotic signaling pathways and by inhibiting STAT3 activity			[110]
Curdione (**117**)		Cytotoxic activity against colorectal cancer; antitumor activity against colorectal cancer	CRC cell model; CRC cell xenograft model in nude mice	Induce ferroptosis in CRC by virtue of m6A methylation			[111]
		Cytotoxic activity against breast cancer; antitumor activity against breast cancer	MCF-7 and MDA-MB-23 cell models; MCF-7 cell xenograft model in nude mice	Inhibit proliferation and induce apoptosis; exert a synergistically inhibitory effect with other chemotherapy drugs through MAPKs and PI3K/AKT pathways			[19]
		Cytotoxic activity against uterine leiomyosarcoma; antitumor activity against uterine leiomyosarcoma	SK-UT-1 and SK-LMS-1 cell models; SK-UT-1 cell xenograft model in nude mice	Decrease the viability and proliferation of SK-UT-1 and SK-LMS-1 cells, improve apoptosis and autophagic death, and exhibit an antitumor effect through indoleamine-2, 3-dioxygenase-1			[112]
		Cytotoxic activity against hepatic carcinoma	HHSEC under the micro-environment of HepG_2_ cells	Inhibit the expressions of VEGF and VEGFR_2_ in HHSECs in HepG_2_ cell micro-environment			[113]
Furanodiene (**139**)		Cytotoxic activity against breast cancer; antitumor activity against breast cancer	MCF-7 and MDA-MB-231 cell models and MCF-7 cell xenograft model in nude mice	Inhibit cell proliferation through apoptosis in a mitochondria-mediated pathway by regulating cyclin D1, CDK2, pRb, and Bcl-2 family proteins; activating caspases and PARP; and the Akt pathway is also be involved			[93]
		Cytotoxic activity against breast cancer	MCF-7 cell model	Inhibit cancer cell growth via the AMPK pathway and induce cell apoptosis via metabolic regulation in chemoresistant MCF-7 breast cancer cells			[94]
		Cytotoxic activity against leukemia	HL60 cell model	Activate bid protein (a substrate of caspase-8), upregulate TNFR1, promote the formation of the TNFR1 complex and the production of TNF-*α* through the activation of TNFR1 signaling pathway, inducing cell apoptosis			[96]
		Cytotoxic activity against lung cancer, breast cancer, leukemia, etc.; antitumor activity against breast cancer	Multi-models	Induce apoptosis in several cancer types by modulating MAPKs/ERK, NF-*κ*B, Akt, and other pathways			[99]
Furanodienone (**140**)		Cytotoxic activity against colorectal cancer; antitumor activity against colorectal cancer	RKO and HT-29 cell models and CRC cell xenograft model in nude mice	Induce G0/G1 arrest and cause apoptosis via the ROS/MAPKs-mediated caspase-dependent pathway			[114]
Zederone (**143**)		Cytotoxic activity against ovarian cancer	SKOV-3 cell model	Inhibit mTOR/p70s6K signaling pathway			[115]
Curcolonol (**186**)	Eudesmane-type sesquiterpenoids	Cytotoxic activity against breast cancer	MDA-MB-231 cell model	Inhibit LIM kinase 1 to downregulate cofilin 1 phosphorylation			[116]
Serralactone A (**205**)		Cytotoxic activity against breast cancer	MDA-MB-231 and MDA-MB-468 cell models	Downregulate LIMK1 activation			[117]
*β*-Elemene (**211**)	Elemane-type sesquiterpenoids	Cytotoxic activity against gastric cancer, hepatocarcinoma, breast cancer, etc.; antitumor activity against hepatocarcinoma, lung cancer, etc.	Multi-models	Inhibit cell proliferation, arrest the cell cycle and induce cell apoptosis; enhance cell immune function associated with malignancy; activate cytoprotective autophagy; reverse multidrug resistance; prevent tumor angiogenesis; enhance the sensitivity of tumor cells to radiotherapy			[101]
		Cytotoxic activity against lung cancer, hepatocarcinoma, breast cancer, etc.; antitumor activity against leukemia, esophageal cancer, gastric cancer, etc.	Multi-models	Inhibit and kill tumor cells through a variety of mechanisms; enhance the effect of radiotherapy or chemotherapy synergistically; regulate autoimmune activity in the treatment of tumors			[100]
*δ*-Elemene (**213**)		Cytotoxic activity against leukemia	HL-60 cell model	Induce apoptosis by activating caspase-3 and interfering with the cell cycle at the G_2_/M phase			[97]
Curzerene (**214**)		Cytotoxic activity against lung carcinoma; antitumor activity against lung carcinoma	SPC-A1 cell model and SPC-A1 cell xenograft model in nude mice	Induce the downregulation of GSTA1 protein and mRNA expression in SPC-A1 cells			[118]
Curzerenone (**215**)		Cytotoxic activity against lung carcinoma	H69AR and MRC5 cell models	Mediate programmed cell death, loss of mitochondrial membrane potential, ROS; and block the ERK/MAPK and NF*-κ*B signaling pathways			[119]
Acomadendrane-4*β*,10*β*-diol (**256**)	Other-type sesquiterpenoids	Cytotoxic activity against colon cancer	RKO cell model	Exhibit antimigratory activity			[65]
Curcumenone (**258**)		Cytotoxic activity against breast cancer	MCF-7 cell model	Exhibit cytotoxic activity	8.3 ± 1.0 μg/mL	0.1 ± 0.0 μg/mL (Doxorubicin)	[105]

### 4.3. Effects on Cardiovascular System

Some of the sesquiterpenoids in *Curcumae Rhizoma* can exert more prominent effects on cardiovascular disease (Table 9), such as anti-platelet aggregation, anti-thrombotic, vasodilation-inducing, and anti-atherosclerotic effects, as well as protective effects against myocardial ischemia–reperfusion injury.

Numerous compounds have been demonstrated to possess anti-thrombotic and anti-platelet activities, with curdione (**117**) emerging as the most potent among them. Fang et al. found that curdione can inhibit thrombin-induced platelet aggregation via regulating the AMP-activated protein kinase-vinculin/talin-integrin *α*IIbβ3 signaling pathway [120]. Furthermore, certain compounds exhibit a specific structure–activity relationship. For instance, the enantiomers (+)-phaeocauline A (**10**) and (−)-phaeocauline A (**11**) exhibited similar activity against arachidonic acid-induced abnormal platelet aggregation. However, their C-4 epimers (+)-phaeocauline B (**12**) and (−)-phaeocauline B (**13**) showed no activity. This indicates that the anti-platelet aggregation activity is stereoselective rather than enantioselective [33].

Beyond the anti-platelet aggregation effects, sesquiterpenoids potentially exert influences on various other aspects of cardiovascular health, including vasodilation, combating atherosclerosis, alleviating cerebral ischemia–reperfusion injury, mitigating myocardial ischemia–reperfusion injury, and intervening with restenosis [1,33,121,122,123,124,125].

In summary, the therapeutic effects on the cardiovascular system are mainly exerted by guaiane-type sesquiterpenoids, including anti-thrombotic, vasodilatory, and anti-atherosclerotic effects, as well as protective effects against cerebral ischemia–reperfusion injury. Certain germacrane-type sesquiterpenoids exhibit notable activity, primarily manifesting as anti-thrombotic effects, with curdione demonstrating particularly pronounced efficacy.

**Table 9:** Effects on cardiovascular system of sesquiterpenoids in *Curcumae Rhizoma*.

Compounds	Compound Types	Activity Types	Pharmacological Models	Effects	Value	Positive Control	Reference
(+)-Phaeocauline A (**10**)	Guaiane-type sesquiterpenoids	Anti-platelet effect	Abnormal platelet aggregation induced by arachidonic acid	Inhibit the platelet aggregation induced by AA	Inhibition rate: 27.78 ± 4.36%	Inhibition rate: 72.89 ± 7.65% (Aspirin)	[33]
(−)-Phaeocauline A (**11**)					Inhibition rate: 31.63 ± 7.10%		
Procurcumenol (**29**)			Platelet aggregation induced by ADP	Inhibit the activity of the MAPK and PI3K/AKT pathways	Inhibition_max_: 76.3%; IC_50_: 0.2560 mg/mL		[8]
Isoprocurcumenol (**43**)					Inhibition_max_: 62.8%; IC_50_: 0.2680 mg/mL		
(+)-Phaeocauline D (**36**)		Vasorelaxant effect	Contraction of rat aortic rings induced by KCl	Exhibit vasorelaxant effects against KCl-induced contraction	Vasorelaxation: 35.51 ± 3.65%		[33]
(−)-Phaeocauline D (**37**)					Maximal vasorelaxation: 38.96 ± 3.26%		
(+)-Phaeocauline E (**41**)					Maximal vasorelaxation: 39.42 ± 4.63%		
(−)-Phaeocauline E (**42**)					Maximal vasorelaxation: 40.93 ± 5.68%		
(+)-Phaeocauline C (**68**)					Maximal vasorelaxation: 47.71 ± 4.35%		
(−)-Phaeocauline C (**69**)					Maximal vasorelaxation: 45.64 ± 6.85%		
Curcumol (**55**)		Protective effect against cardiac remodeling	Isoproterenol (ISO)-induced cardiac remodeling	Attenuate cardiac dysfunction, myocardial fibrosis, and hypertrophy; inhibit the inflammation and apoptosis induced by ISO and TGF-*β*1; inhibit the AKT/NF-*κ*B pathway			[126]
Zedoarondiol (**19**)		Protective effect against ox-LDL-induced injury of endothelial cells	ox-LDL-induced endothelial cell injury	Inhibit oxidative stress and inflammation via the Nrf2/HO-1 pathway			[121]
		Anti-atherosclerosis effect	Arteriosclerosis in apoE mice induced by high-fat diet; THP-1 monocyte migration and adhesion experience	Ameliorate AS plaque and inhibit monocyte migration and adhesion to endothelial cells via regulating the CXCL12/CXCR4 pathway			[122]
			Arteriosclerosis in apoE mice induced by high-fat diet	Inhibit aortic plaque, inhibit the expressions of HIF 1*α* and downstream protein VEGF, and alleviate oxidative stress injury			[123]
		Anti-atherosclerosis effect, intervene in-sent restenosis effect	PDGF-BB-induced VSMCs proliferation	Inhibit PDGF-BB-induced VSMCs proliferation via AMPK-mediated downregulation of the mTOR/p70S6K pathway and upregulation of the p53/p21 pathway			[124]
		Protective effect against coronary heart disease and cardiovascular events	RAW264.7 macrophage inflammation model	Regulate the expression of Sirt1 of the target gene of miRNA-34a and the downstream inflammatory pathway			[125]
Germacrone (**116**)	Germacrane-type sesquiterpenoids	Protective effect against cardiac remodeling	Isoproterenol-induced mouse model; isoproterenol-induced neonatal rat cardiomyocytes	Attenuate oxidative stress, inflammation, and apoptosis in cardiac remodeling by inhibiting the PI3K/AKT pathway			[127]
		Protective effect against cerebral ischemia/reperfusion injury	Cerebral ischemia–reperfusion injury model in rats	Increase the levels of Bcl-2 and inhibit the levels of caspase-3 and Bax; induce Akt activation			[128]
Curdione (**117**)		Neuroprotective effects against focal cerebral ischemia reperfusion injury in rats	Cerebral ischemia–reperfusion injury model in rats	Reduce infarct size and neurological deficits, promote cognitive function recovery and recover neuronal morphologic damage; block the increase in MDA content and elevate the activities of SOD, CAT, and GSH-PX; increase the Bcl-2/Bax ratio and decrease cellular apoptosis			[1]
		Anti-platelet aggregation effect	Thrombin-induced platelet aggregation	Regulate the AMP-activated protein kinase-vinculin/talin-integrin αIIbβ3 signaling pathway			[120]
			Platelet aggregation induced by ADP	Inhibit the activity of MAPK and PI3K/AKT pathways	Inhibition_max_: 85.6%; IC_50_: 0.1611 mg/mL		[8]
			Platelet aggregation induced by thrombin, PAF, ADP, AA, and tail thrombosis models	Increase cAMP levels, inhibit intracellular Ca^2+^ mobilization, and increase vasodilation			[13]
Neocurdione (**118**)			Platelet aggregation induced by ADP	Inhibit the activity of the MAPK and PI3K/AKT pathways	Inhibition_max_: 77.6%; IC_50_: 0.2290 mg/mL		[8]
(1*R*,4*S*,5*R*,9*R*,10*S*)-9-Hydroxy-zederone epoxide (**145**)			Platelet aggregation induced by ADP and AA	Inhibit the platelet aggregation induced by ADP and AA	Inhibition_max_: 21.07 ± 8.67%; 27.73 ± 6.42%	Inhibition_max_: 44.83 ± 1.24%; 72.74 ± 7.54% (Aspirin)	[59]
*β*-Elemene (**211**)	Elemane-type sesquiterpenoids	Anti-thrombotic effect	Anticoagulant experiment and plasma recalcificatic time in wistar rabbits, acute blood-stasis rat model made by using ice-cold water, platelet aggregation induced by ADP and AA	Dissolve the thrombus and blood clots, prolong prothrombin and thrombin times, inhibit platelet aggregation			[129]
		Anti-atherosclerosis effect	Arteriosclerosis in apoE mice induced by high-fat diet; HUVEC cell model	Increase the levels of plasma NO_2_/NO_3_, increase the expression of phosphorylation-eNOS; upregulate the Akt/eNOS signaling pathway and NO production in HUVECs			[130]
Curcumadione (**269**)	Other-type sesquiterpenoids	Anti-platelet effect	Platelet aggregation induced by ADP	Inhibit the activity of MAPK and PI3K/AKT pathways	Inhibition_max_: 76.3%; IC_50_: 0.2560 mg/mL		[8]

### 4.4. Hepatoprotective Activity

Modern research has revealed that many compounds in *Curcumae Rhizoma* have hepatoprotective activity, which aligns with the traditional belief that *Curcumae Rhizoma* benefits the liver (Table 10). These compounds markedly attenuate the oxidative damage induced by H_2_O_2_ in LO_2_ cells and induce HepG_2_ apoptosis to play a hepatoprotective role [18]. In addition, certain compounds demonstrate a protective effect against acute liver injury induced by D-galactosamine (D-GalN)/LPS and inhibit D-GalN-induced cytotoxicity. Interestingly, several sesquiterpenoids are found to strengthen the cytotoxicity induced by D-GalN, even though they show little cytotoxic effect on the hepatocytes in the absence of D-GalN, such as zedoarondiol (**18**), aerugidiol (**31**), isocurcumenol (**60**), and curcumenone (**258**). Actually, this phenomenon exhibits structural relevance rather than concentration dependence, as germacrane-type sesquiterpenoids are prone to exert inhibition, while guaiane-type sesquiterpenoids tend to strengthen the effect [4,131]. Several compounds manifest hepatoprotective, anti-fibrotic, and anti-fatty liver effects through mechanisms encompassing cytotoxic activity, choleretic properties, and ameliorating liver fibrosis and the modulation of sinusoidal capillarization [3,9,80,132,133,134]. In particular, *β*-elemene (**211**) in the volatile oils of Ezhu has been developed into an injection, which has been approved by the state for antitumor drugs and has been widely applied in hepatoma treatment. Some studies have found that Ezhu exhibits certain hepatotoxicity; at a high dosage, Ezhu can obviously decrease hepatocytic activity, even aggravating liver injury. It has been shown that the maximum tolerable dose in experimental mice is 224 g crude drug/kg of Ezhu medicinal material. Moreover, several compounds of Ezhu, including germacrone (**116**), curdione (**117**), and furanodiene (**139**), are found to have both hepatoprotective and hepatocytotoxic effects, implying that the use of these drugs carries risks. Germacrone (**116**) exerts effects at non-toxic concentrations (30 μM) but leads to alterations in cholesterol and lipid metabolism at slightly toxic (100 μM) and toxic concentrations (250 μM) [9].

In summary, the material basis of hepatoprotective activity mainly comprises guaiane-type, germacrane-type, as well as individual other types of sesquiterpenoids. Among them, *β*-elemene, which is an elemane-type sesquiterpenoid, mainly exerts its activity through protecting against liver injury, ameliorating hepatic fibrosis, exerting antitumor effects, and stimulating bile flow into the duodenum. Most of these active ingredients are shared by two or three medicinal sources.

### 4.5. Anti-Diabetic Activity

Diabetes is the third most prevalent chronic ailment in China, following cardiovascular disease and oncological conditions, with its incidence steadily rising each year. Sesquiterpenoids from *Curcumae Rhizoma* have been found to exert anti-diabetic effects by increasing glucose consumption [31], improving insulin signaling and glucose circulation [138], accelerating pre-adipocyte differentiation [139], and inhibiting fatty acid synthesis and uptake (Table 11) [80]. In addition, some of the compounds improve diabetic retinopathy and also reduce diabetic retinal vascular exudation and leakage [81,140].

In conclusion, germacrane-type, guaiane-type, and other types of sesquiterpenoids in *Curcumae Rhizoma* demonstrate predominant anti-diabetic properties. These sesquiterpenoids are evenly distributed across the three plants, with a significant proportion being shared compounds among two or three medicinal herbs.

### 4.6. Other Biological Activities

In addition to the above activities, it has been found that sesquiterpenoids in *Curcumae Rhizoma* have a variety of other biological activities, including antioxidant, anti-microbial, anti-viral, skin regeneration, anti-aging, neuroprotective, and anti-septic effects (Table 12). The research findings indicate that wenyujinin Q (**17**), zedoarondiol (**18**), isozedoarondiol (**20**), phaeocaulisin E (**21**), procurcumadiol (**33**), neoprocurcumenol (**35**), and various other compounds show extensive antibacterial effects and antifungal properties [36,40]. Notably, certain compounds exhibit noteworthy efficacy against both influenza A and influenza B viruses [29]. Regarding the antioxidant potential, a diverse array of sesquiterpenoids manifest noteworthy antioxidant properties and the efficacious scavenging of free radicals. Studies have elucidated the involvement of specific compounds, such as germacrone (**116**), in diverse oxidative stress models. These compounds actively diminish free radical concentrations within the organism, alleviate oxidative harm, and consequently hold promise in the prevention and treatment of associated diseases [80]. In the context of skin regeneration, it has been discovered that alismoxide (**3**), isozedoarondiol (**20**), isoprocurcumenol (**43**), germacrone (**116**), and 13-hydroxygermacrone (**124**) can activate the epidermal growth factor receptor, thereby promoting skin regeneration [142,143,144]. In relation to other activities, procurcumenol (**29**), germacrone (**116**), and dehydrocurdione (**121**) also exhibit neuroprotective effects [143]; curcumanolide A (**241**) has a relaxant effect on uterine smooth muscle tissue [15]; curdione (**117**) attenuates sepsis-induced lung injury [145]; curcumenol (**61**) reduces disc inflammation and improves disc catabolism [146]; zederone (**143**) has the potential to be a drug for the treatment of dementia [147]; and curcumenone (**258**) can exert a protective effect against intoxication [148]. These activities are primarily attributed to guaiane-type, germacrane-type, eudesmane-type, and elemane-type sesquiterpenoids.

## 5. Conclusions

*Curcumae Rhizoma*, a crucial medicinal herb, has a long history of medicinal use and exhibits remarkable therapeutic efficacy. Research on the chemical composition and pharmacological activities of this medicine has been extensively conducted both in China and internationally. The primary chemical constituents identified include curcumins and sesquiterpenoids. The traditional utilization of *Curcumae Rhizoma* among communities exhibits a lack of systematicity, with medicinal sources presenting notable diversity. Therefore, this article provides an extensive review of sesquiterpenoids isolated from the rhizomes of *C. phaeocaulis*, *C. kwangsiensis*, and *C. wenyujin*, primarily based on the Chinese Pharmacopeia. A total of 279 sesquiterpenoids have been reported in the relevant literature, showcasing an extensive structural diversity comprising many analogs, enantiomers, diastereomers, and geometric isomers. These compounds encompass diverse types, featuring guaiane-type, germacrane-type, eudesmane-type, elemane-type, and cadinane-type sesquiterpenoids. A total of 79 sesquiterpene compounds were obtained from *C.kwangsiensis*, 167 from *C. wenyujin*, and 143 from *C. phaeocaulis*. It was found that all three plants were dominated by guaiane-type and germacrane-type sesquiterpenoids, and some compounds were present in all three plants at the same time, with 14 shared compounds among the guaiane-type sesquiterpenoids and 12 among the germacrane-type sesquiterpenoids. These findings demonstrate that all three sources can be utilized as substitutes for *Curcumae Rhizoma*, notwithstanding their diverse botanical origins.

Pharmacological studies have revealed that all types of sesquiterpenoids possess some anti-inflammatory properties, while their cancer-related activity is concentrated in guaiane-type, germacrane-type, and elemane-type sesquiterpenoids. For the treatment of cardiovascular diseases, guaiane-type and germacrane-type sesquiterpenoids are mainly involved, of which zedoarondiol has a significant anti-atherosclerotic effect and curdione has a significant anti-thrombotic effect. The hepatoprotective and anti-diabetic effects are also predominantly concentrated in the guaiane-type and germacrane-type sesquiterpenoids. In terms of compound types, guaiane-type and germacrane-type sesquiterpenoids were found to include a variety of active substances. Eudesmane-type sesquiterpenoids are among the material bases for anti-inflammatory activity, while elemane-type sesquiterpenoids are mostly associated with significant cancer-related effects. Most of the active monomers in them are present in two or three medicinal herbs. Some of the compounds common to all three plants, such as zedoarondiol, isozedoarondiol, curcumol, curcumenol, curdione, furanodiene, zederone, *β*-elemene, curzerene, and curzerenone, are found at high levels in *Curcumae Rhizoma*, and they have a wide range of activities and high therapeutic efficacy, which also supports the scientific validity of the use of the three herbs together as *Curcumae Rhizoma*.

Various studies have shown that the sesquiterpenoids in *Curcumae Rhizoma* have immense potential as new drug sources, but there are still barriers to their use. To address these limitations, future research should focus on several areas: (1) at present, sesquiterpenoids are mainly extracted by means of organic solvent extraction and steam distillation, followed by further purification by column chromatography, including silica gel column chromatography, reversed-phase C18 silica gel column chromatography, Sephadex LH-20 column chromatography, ODS column chromatography, HPLC, and preparative TLC. Contemporary isolation methods are relatively mature, leading to a diverse array of compounds. However, due to the volatile and unstable nature of the sesquiterpenoids, the extant separation methodologies encounter certain constraints. Thus, the imperative arises to embrace innovative technological paradigms to surmount these challenges. For instance, separation can be conducted in a closed system at room temperature using HPLC-SPE, which gradually emerges as an indispensable solution to address these limitations. (2) Guaiane-type and germacrane-type sesquiterpenoids possess more prominent bioactivities compared to other sesquiterpenoids. However, the isolation methods currently in common use are not specific for obtaining different types of sesquiterpenoids. Therefore, it is crucial to implement novel methods, techniques, and strategies, including Global Natural Products Social (GNPS) molecular networking, Small Molecule Accurate Recognition Technology (SMART), and high-sensitivity LC-MS/MS, to achieve the precise and targeted identification of specific compounds. (3) The activity of sesquiterpenoids within *Curcumae Rhizoma* is rich and diverse, oriented towards anti-inflammatory, antitumor, cytotoxic, anti-cardiovascular disease, and hepatoprotective effects. Several compounds have been used for the development of new drugs, showing that this herb possesses great potential as a new drug source. Thus, there is a need to broaden the research on pharmacological activity and expand the study on the underlying mechanisms.

## Figures and Tables

**Table 3:** Eudesmane-type sesquiterpenoids of *Curcumae Rhizoma*.

No.	Compounds	Medicinal Source	Reference
**170**	Phaeocaulistriol A	*C. phaeocaulis*	[28]
**171**	Phaeocaulistriol B	*C. phaeocaulis*	[28]
**172**	1*α*,4*β*-Dihydroxyeudesm-7(11)-en-8-one	*C. phaeocaulis*, *C. kwangsiensis*	[2,38]
**173**	1-Hydroxyeudesma-4(14),7(11)-dien-8-one	*C. phaeocaulis*	[2]
**174**	1-Hydroxyeudesma-3,7(11)-dien-8-one	*C. phaeocaulis*	[2]
**175**	9-Hydroxyeudesma-3,7(11)-dien-6-one	*C. phaeocaulis*	[2]
**176**	Phaeusmane A	*C. phaeocaulis*	[2]
**177**	Phaeusmane B	*C. phaeocaulis*	[2]
**178**	Phaeusmane D	*C. phaeocaulis*	[2]
**179**	Phaeusmane E	*C. phaeocaulis*	[2]
**180**	Phaeusmane C	*C. phaeocaulis*	[2]
**181**	Eudesm-11-ene-4*α*,6*α*-diol	*C. phaeocaulis*, *C. kwangsiensis*	[2,12]
**182**	Capillosanane Z	*C. wenyujin*	[18]
**183**	Cyperusol C	*C. wenyujin*, *C. phaeocaulis*	[2,31]
**184**	1*β*-Hydroxyeudesma-4,11-dien-3-one	*C. phaeocaulis*	[2]
**185**	Zedoarofuran	*C. phaeocaulis*	[37]
**186**	Curcolonol	*C. wenyujin*, *C. phaeocaulis*	[2,66]
**187**	9*α*-Hydroxycurcolonol	*C. phaeocaulis*	[56]
**188**	(+)-Phaeocauline G	*C. phaeocaulis*	[33]
**189**	(−)-Phaeocauline G	*C. phaeocaulis*	[33]
**190**	Curcodione	*C. wenyujin*, *C. phaeocaulis*	[2,66]
**191**	4*α*-Hydroxy-8,12-epoxyeudesma-7,11-diene-1,6-dione	*C. phaeocaulis*	[37]
**192**	Curcolone	*C. phaeocaulis*	[2]
**193**	3*α*-Hydroxy-4-deoxy-5-dehydrocurcolonol	*C. phaeocaulis*	[56]
**194**	Chlorantene D	*C. phaeocaulis*	[28]
**195**	Chlomultin B	*C. phaeocaulis*	[2]
**196**	Myrrhterpenoid N	*C. phaeocaulis*	[2]
**197**	Phaeusmane F	*C. phaeocaulis*	[2]
**198**	Phaeusmane G	*C. phaeocaulis*	[2]
**199**	1*β*,8*β*-Dihydroxyeudesma-4,7(11)-dien-8*α*,12-olide	*C. phaeocaulis*	[2]
**200**	(7Z)-1*β*,4*α*-Dihydroxy-5*α*,8*β*(H)-eudesm-7(11)-en-8,12-olide	*C. phaeocaulis*, *C. wenyujin*	[2,66]
**201**	(7*Z*)-1*β*,4*β*-Dihydroxy-5*α*,8*β*(H)-eudesm-7(11)-en-8,12-olide	*C. phaeocaulis*, *C. wenyujin*	[2,32]
**202**	Curcolide	*C. wenyujin*	[29,66]
**203**	Wenyujinlactone A	*C. wenyujin*	[67]
**204**	1*β*,8*β*-Dihydroxyeudesma-3,7(11)-dien-8*α*,12-olide	*C. phaeocaulis*	[2]
**205**	Serralactone A	*C. phaeocaulis*	[2,56]
**206**	Hydroxyatractylolide	*C. kwangsiensis*	[60]
**207**	Butenolide III	*C. wenyujin*	[68]
**208**	Neolitacumone A	*C. phaeocaulis*, *C. wenyujin*	[2,37,67]
**209**	Phaeusmane I	*C. phaeocaulis*	[69]
**210**	Phaeusmane H	*C. phaeocaulis*	[2]

**Table 4:** Elemane-type sesquiterpenoids of *Curcumae Rhizoma*.

No.	Compounds	Medicinal Source	Reference
**211**	*β*-Elemene	*C. wenyujin*, *C. phaeocaulis*, *C. kwangsiensis*	[70,71,72]
**212**	*γ*-Elemene	*C. wenyujin*, *C. phaeocaulis*, *C. kwangsiensis*	[70,71,72]
**213**	*δ*-Elemene	*C. wenyujin*, *C. phaeocaulis*, *C. kwangsiensis*	[70,71,72]
**214**	Curzerene	*C. wenyujin*, *C. phaeocaulis*, *C. kwangsiensis*	[26]
**215**	Curzerenone	*C. wenyujin*, *C. phaeocaulis*, *C. kwangsiensis*	[31,38,57]
**216**	Epicurzerenone	*C. phaeocaulis*	[73]
**217**	Isogermafurenolide	*C. wenyujin*	[44,46]
**218**	5-Isopropenyl-3,6-dimethyl-6-vinyl-5,6,7,7*α*-tetrahydro-4H-benzofuran-2-one	*C. wenyujin*	[58]
**219**	8*β*-Hydroxy-isogermafureolide	*C. wenyujin*, *C. phaeocaulis*, *C. kwangsiensis*	[12,56,68]
**220**	Hydroxyisogermafurenolide	*C. wenyujin*, *C. kwangsiensis*	[44,46,52,58,66]
**221**	5*β*H-Elema-1,3,7,8-tetraen-8,12-olide	*C. wenyujin*	[44]
**222**	8*β*-Methoxy-isogermafurenolide	*C. phaeocaulis*	[69]
**223**	8*β*(H)-Elema-1,3,7(11),8-tetraen-8,12-lactam	*C. wenyujin*, *C. phaeocaulis*	[36,46,69]
**224**	8*β*(H)-Elema-1,3,7(11)-trien-8,12-lactam	*C. phaeocaulis*	[56]

**Table 5:** Cadinane-type sesquiterpenoids of *Curcumae Rhizoma*.

No.	Compounds	Medicinal Source	Reference
**225**	Wenyujinone F	*C. wenyujin*	[18]
**226**	Wenyujinone E	*C. wenyujin*	[18]
**227**	7-Hydroxy-5(10),6,8-cadinatriene-4-one	*C. wenyujin*	[29]
**228**	Phacadinane B	*C. phaeocaulis*	[37,74]
**229**	Phacadinane A	*C. phaeocaulis*	[74]
**230**	Curcujinone B	*C. wenyujin*	[31]
**231**	(+)-Commyrrin A	*C. wenyujin*, *C. kwangsiensis*	[18,38]
**232**	(−)-Commyrrin A	*C. wenyujin*, *C. kwangsiensis*	[38]
**233**	Pyrocurzerenone	*C. kwangsiensis*	[38]
**234**	Furanocadalene	*C. kwangsiensis*	[38]
**235**	Curcujinone A	*C. wenyujin*	[31]
**236**	Phacadinane C	*C. phaeocaulis*	[74]
**237**	Phacadinane D	*C. phaeocaulis*, *C. kwangsiensis*	[12,56,74]
**238**	4,5-Seco-pyrocurzerenone	*C. kwangsiensis*	[38]

**Table 6:** Other-type sesquiterpenoids of *Curcumae Rhizoma*.

No.	Compounds	Medicinal Source	Reference
**239**	Curcumalactone	*C. wenyujin*	[29,31,43]
**240**	7-Epicurcumalactone	*C. wenyujin*	[68]
**241**	Curcumanolide A	*C. wenyujin*, *C. phaeocaulis*	[15,31,45]
**242**	Curcumanolide B	*C. wenyujin*	[31,45]
**243**	(+)-Phaeocauline F	*C. phaeocaulis*	[33]
**244**	(−)-Phaeocauline F	*C. phaeocaulis*	[33]
**245**	Phaeocaudione	*C. phaeocaulis*	[69]
**246**	Phaeocauone	*C. phaeocaulis*, *wenyujin*	[32,69]
**247**	Wenyujinin L	*C. wenyujin*, *C. phaeocaulis*	[35,37]
**248**	Wenyujinol P	*C. wenyujin*	[32]
**249**	Curcumolide	*C. wenyujin*, *C. kwangsiensis*	[12,45]
**250**	Gajutsulactone A	*C. wenyujin*	[31]
**251**	Gajutsulactone B	*C. wenyujin*	[31]
**252**	Wenyujinin C	*C. wenyujin*	[31,35]
**253**	Wenyujinin D	*C. wenyujin*	[35]
**254**	Wenyujinin E	*C. wenyujin*	[35,36]
**255**	Phasalvione	*C. phaeocaulis*, *C. wenyujin*	[28,69]
**256**	Acomadendrane-4*β*,10*β*-diol	*C. kwangsiensis*	[65]
**257**	(4*S*)-Dihydrocurcumenone	*C. wenyujin*	[66]
**258**	Curcumenone	*C. wenyujin*	[44,46,66]
**259**	7*α*,11-Epoxy-6*α*-hydroxy-carabrane-4,8-dione	*C. wenyujin*	[44,68]
**260**	4,8-Dioxo-6*β*-methoxyl-7*α*,11-epoxycarabrane	*C. wenyujin*	[40]
**261**	4,8-Dioxo-6*β*-methoxyl-7*β*,11-epoxycarabrane	*C. wenyujin*	[40]
**262**	4,8-Dioxo-6*β*-hydroxyl-7*β*,11-epoxycarabrane	*C. wenyujin*	[36]
**263**	4,8-Dioxo-6*β*-hydroxyl-7*α*,11-epoxycarabrane	*C. wenyujin*	[36,40]
**264**	7*α*,11-Epoxy-6*α*-methoxy-carabrane-4,8-dione	*C. wenyujin*	[44]
**265**	Curcumenolactone A	*C. phaeocaulis*	[37]
**266**	Curcumenolactone B	*C. phaeocaulis*	[37]
**267**	8,11-Epidioxy-8-hydroxy-4-oxo-6-carabren	*C. wenyujin*	[44]
**268**	Wenyujindiol A	*C. wenyujin*	[61]
**269**	Curcumadione	*C. wenyujin*	[63]
**270**	Curcumadionol	*C. wenyujin*, *C. phaeocaulis*	[16,66]
**271**	(6*R*)-Dehydroxysipanolinolide	*C. wenyujin*	[66]
**272**	Wenyujinone H	*C. wenyujin*	[18]
**273**	Wenyujinone I	*C. wenyujin*	[18]
**274**	*αr*-Turmerone	*C. phaeocaulis*	[57]

**Table 10:** Hepatoprotective of sesquiterpenoids in *Curcumae Rhizoma*.

Compound	Compound Types	Activity Types	Pharmacological Models	Effects	Value	Positive Control	Reference
Zedoarondiol (**18**)	Guaiane-type sesquiterpenoids	Hepatoprotective effect	D-GalN/LPS-induced liver injury	Show a potent protective effect on D-GaIN/LPS-induced acute liver injury	Liver injury: 60.7 ± 10.5%, 54.7 ± 12.7%	Liver injury: 99.0 ± 0.1%, 98.3 ± 0.0% (Hydrocortisone)	[4,131]
Aerugidiol (**31**)					Liver injury: 88.0 ± 2.0%, 89.1 ± 0.7%		
Isocurcumenol (**60**)					Liver injury: 77.3 ± 6.6%, 80.2 ± 5.5%		
Curcumenol (**61**)			D-GalN/LPS-induced liver injury; D-GalN-induced cytotoxicity	Show a potent protective effect on D-GaIN/LPS-induced acute liver injury; inhibit D-GalN-induced cytotoxicity	Liver injury: 50.7 ± 13.8%, 53.4 ± 13.4%; hepatocytotoxicity: 25.1 ± 5.3%		
Curcumol (**55**)		Anti-liver fibrosis effect	LSECs accompanied by an abnormal angioarchitecture; liver fibrosis rats induced by CCl_4_	Attenuate liver sinusoidal endothelial cell angiogenesis via regulating Glis-PROX1-HIF-1*α* in liver fibrosis			[132]
			Liver fibrosis rats induced by CCl_4_; HSCs and LX-2 cell models	Target RIPK1/RIPK3 complex-dependent necroptosis via JNK1/2-ROS signaling for the treatment of hepatic fibrosis			[133]
			HSC cell model	Promote autophagy in HSCs, mediate the degradation of NCOA4 and FTH1 complexes, release iron ions, lead to iron overload, and induce ferroptosis			[134]
		Anti-hepatobiliary disease effect	Liver fibrosis rats induced by CCl_4_; HSCs, HepG_2_, and RBE cell models	Inhibit the activity of RhoROCK and MAPK signaling pathways, inhibit HSC migration and adhesion, and inhibit cell proliferation			[9]
Germacrone (**116**)	Germacrane-type sesquiterpenoids	Anti-liver fibrosis effect	LX-2 and LO_2_ cell models	Reduce ROS release to avoid liver injury-induced HSC activation; inhibit the activation and survival of HSCs by regulating TGF-beta/Smad and apoptosis pathways			[135]
			Liver fibrosis rats induced by CCl_4_; LX-2 cell model	Attenuate hepatic fibrosis via the PI3K/AKT/mTOR signaling pathway			[136]
		Anti-hepatoma effect	HepG_2_ and Bel7402 cell models	Regulate the expression of proteins related to the G2/M cell cycle, apoptosis and p53; oxidative damage may be involved			[3]
		Hepatoprotective effect	D-GalN/LPS-induced liver injury; D-GalN-induced cytotoxicity	Show potent protective effect on D-GaIN/LPS-induced acute liver injury; inhibit D-GalN-induced cytotoxicity	Liver injury: 82.9 ± 5.4%, 78.1 ± 6.8%; hepatocytotoxicity: 59.8 ± 6.3%	Liver injury: 99.0 ± 0.1%, 98.3 ± 0.0% (Hydrocortisone)	[4,131]
Curdione (**117**)		Hepatoprotective effect	D-GalN/LPS-induced liver injury; D-GalN-induced cytotoxicity	Show potent protective effect on D-GaIN/LPS-induced acute liver injury; inhibit D-GalN-induced cytotoxicity	Liver injury: 76.6 ± 4,7%, 74.6 ± 4.7%; hepatocytotoxicity: 77.1 ± 5.8%	Liver injury: 99.0 ± 0.1%, 98.3 ± 0.0% (Hydrocortisone)	[4,131]
Neocurdione (**118**)					Liver injury: 59.3 ± 10.6%, 58.4 ± 11.1%; hepatocytotoxicity: 44.6 ± 5.3%		
Wenyujinone D (**120**)			Oxidative damage induced by H_2_O_2_ in LO_2_ cells	Weaken the oxidative damage induced by H_2_O_2_ in LO_2_ cells via strengthening cell viability	Cell viability: 63.6% (H_2_O_2_: 50.7%)		[18]
Wenyujinone B (**162**)					Cell viability: 86.0% (H_2_O_2_: 50.7%)		
Furanodiene (**139**)		Hepatoprotective effect	Oxidative damage induced by H_2_O_2_ in LO_2_ cells	Weaken the oxidative damage induced via strengthening cell viability	Cell viability: 85.0% (H_2_O_2_: 50.7%)		[18]
			D-GalN/LPS-induced liver injury	Show potent protective effect on D-GaIN/LPS-induced acute liver injury	Liver injury: 72.9 ± 6.7%, 74.3 ± 5.7%	Liver injury: 99.0 ± 0.1%, 98.3 ± 0.0% (Hydrocortisone)	[131]
		Anti-hepatoma effect	HepG_2_ cell model	Induce G_2_/M cell cycle arrest and apoptosis through MAPK signaling and mitochondria-caspase pathway in HepG_2_ cells			[137]
		Anti-hepatobiliary disease effect	HepG_2_ cell model	Induce G_2_/M cell cycle arrest and apoptosis			[9]
*β*-Elemene (**211**)	Elemane-type sesquiterpenoids	Hepatoprotective effect; anti-fibrotic effect; anti-hepatoma effect	Liver fibrosis rats induced by CCl_4_; HSC-T6, HepG_2_, BNL, and H22 cell models	Inhibit the biological effect of ANG II and delayed liver fibrosis; inhibit cell migration and invasion through TGF-*β*1/Smad, JNK1/2-ROS, NF-*κ*B, and other pathways			[9]
Curcumenone (**258**)	Other-type sesquiterpenoids	Hepatoprotective effect	D-GalN/LPS-induced liver injury	Show potent protective effect on D-GaIN/LPS-induced acute liver injury	Liver injury: 90.1 ± 0.5%, 88.0 ± 0.4%	Liver injury: 99.0 ± 0.1%, 98.3 ± 0.0% (Hydrocortisone)	[131]
Curcumenolactone A (**265**)			D-GalN-induced cytotoxicity	Inhibit D-GalN-induced cytotoxicity	Inhibition: 65.5 ± 5.7% (100 μM)		[4]
Curcumenolactone B (**266**)					Inhibition: 71.1 ± 4.3% (100 μM)		

**Table 11:** Anti-diabetic effects of sesquiterpenoids in *Curcumae Rhizoma*.

Compounds	Compound Types	Activity Types	Pharmacological Models	Effects	Value	Positive Control	Ref.
4,10-Epizedoarondiol (**25**)	Guaiane-type sesquiterpenoids	Anti-diabetic effect	PTP1B inhibitory assay	Inhibit the activity of PTP1B	IC_50_: 35.1 μM	IC_50_: 5.62 μM (RK-682); 2.75 μM (Ulsolic acid)	[138]
Procurcumenol (**29**)					IC_50_: 45.6 μM		
Aerugidiol (**31**)					IC_50_: 35.7 μM		
Alismoxide (**3**)			Type 2 diabetes mellitus mouse model induced by combined administration of streptozotocin and nicotinamide	Accelerate 3T3-L1 pre-adipocyte differentiation and possess a hypoglycemic property			[139]
7*α*,11*α*-Epoxy-5*β*-hydroxy-9-guaiane-8-one (**46**)			Glucose transportation model on HepG_2_ cells	Increase glucose consumption in HepG_2_ cells	46.1% (10 μM)		[31]
Curcumenol (**61**)					47.0% (10 μM)		[31]
Curdione (**117**)	Germacrane-type sesquiterpenoids	Anti-diabetic effect	Glucose transportation model on HepG_2_ cells	Increase glucose consumption in HepG_2_ cells	74.0% (10 μM)		[31]
Zederone (**143**)					57.0% (10 μM)		[31]
Heyneanone C (**122**)			PTP1B Inhibitory Assay	Inhibit the activity of PTP1B	IC_50_: 35.2 μM	IC_50_: 5.62 μM (RK-682); 2.75 μM (Ulsolic acid)	[138]
Germacrone (**116**)		Regulation of glucose–lipid metabolism	Multi-models	Regulate adipogenesis, lipolysis, and AMPKα pathway; inhibit fatty acid synthesis and uptake by suppressing the activation of the SREBP signaling pathway to alleviate hyperlipidemia and stimulate FA-*β* oxidation to improve lipid metabolism			[80]
8*β*(H)-Elema-1,3,7(11),8-tetraen-8,12-lactam (**223**)	Elemane-type sesquiterpenoids	Attenuate ischemia-induced retinal neovascularization effect	Diabetic retinopathy rat models	Exert anti-inflammatory and anti-angiogenic effects through inhibiting NF-*κ*B and VEGFR2 signaling pathways; reduce retinal microvascular leakage; induce retinal neovascularization			[141]
Gajutsulactone B (**251**)	Other-type sesquiterpenoids	Anti-diabetic effect	Glucose transportation model on HepG_2_ cells	Increase glucose consumption in HepG_2_ cells	47.2% (10 μM)		[31]
Wenyujinin C (**252**)					49.7% (10 μM)		
Curcumolide (**249**)		Attenuate diabetic retinopathy effect	STZ-induced diabetic rat model and TNF-α-stimulated HUVECs	Reduce diabetic retinal vascular leukostasis and leakage partly via the inhibition of the p38MAPK/NF-*κ*B signaling pathway			[81]
		Attenuate ischemia-induced retinal neovascularization effect	HUVEC cell model; oxygen-induced mouse retinopathy model	Exert anti-angiogenic activity and attenuate ischemia-induced retinal neovascularization via the VEGFR2 signaling pathway			[140]

**Table 12:** Other biological activities of sesquiterpenoids in *Curcumae Rhizoma*.

Compounds	Compound Types	Activity Types	Pharmacological Models	Effects	Value	Positive Control	Reference
Wenyujinin Q (**17**)	Guaiane-type sesquiterpenoids	Antifungal activity	Broad-spectrum antifungal activities	Exhibit broad-spectrum antifungal activities	*A. brassicicola*: 50 μg/mL; *P. parasitica var. nicotianae*: 100 μg/mL; *C. capsici*: 50 μg/mL; *B. oryzae*: 50 μg/mL; *D. medusaea Nitschke*: 100 μg/mL; *C. paradoxa Moreau*: 50 μg/mL; *E. turcicum*: 25 μg/mL; *P. theae*: 25 μg/mL; *A. citri*: 100 μg/mL	*A. brassicicola*: 12.5 μg/mL; *P. parasitica var. nicotianae*: 50 μg/mL; *C. capsici*: 12.5 μg/mL; *B. oryzae*: 50 μg/mL; *D. medusaea Nitschke*: 50 μg/mL; *C. paradoxa Moreau*: 25 μg/mL; *E. turcicum*: 12.5 μg/mL; *P. theae*: 25 μg/mL; *A. citri*: 25 μg/mL (Prochloraz)	[36]
Phaeocaulisin E (**21**)					*A. brassicicola*: 100 μg/mL; *P. parasitica var. nicotianae*: 50 μg/mL; *C. capsici*: 50 μg/mL; *B. oryzae*: 100 μg/mL; *D. medusaea Nitschke*: 100 μg/mL; *C. paradoxa Moreau*: 25 μg/mL; *E. turcicum*: 50 μg/mL; *P. theae*: 25 μg/mL; *A. citri*: 50 μg/mL		
Neoprocurcumenol (**35**)					*A. brassicicola*: 12.5 μg/mL; *P. parasitica var. nicotianae*: 50 μg/mL; *C. capsici*: 25 μg/mL; *B. oryzae*: 50 μg/mL; *D. medusaea Nitschke*: 100 μg/mL; *C. paradoxa Moreau*: 25 μg/mL; *E. turcicum*: 50 μg/mL; *P. theae*: 25 μg/mL; *A. citri*: 50 μg/mL		
Procurcumadiol (**33**)		Antibacterial effect	Antibacterial activity against *E. coli*	Antibacterial activity against *E. coli*	*E. coli*: 1.25 μg/mL	*E. coli*: 0.3 μg/mL (Ciprofloxacin)	[40]
7*β*,8*α*-Dihydroxy-1*α*,4*α*H-guai-10(15)-en-5*β*,8*β*-endoxide (**57**)		Anti-viral activity	Influenza virus A	Show anti-viral activity against the influenza virus A	IC_50_: 9.18 ± 0.46 μM	IC_50_: 8.06 ± 0.64 μM (Ribavirin); 47.42 ± 1.96μM (Oseltamivir)	[29]
1*α*,8*α*-Epidioxy-4*α*-hydroxy-5*α*H-guai-7(11),9-dien-12,8-olide (**110**)					IC_50_: 6.80 ± 0.13 μM	IC_50_: 8.06 ± 0.64 μM (Ribavirin); 47.42 ± 1.96μM (Oseltamivir)	
9-Oxo-neoprocurcumenol (**45**)		Antioxidant property	Nrf2-luciferase activity in HEK 293 cells	Exhibit antioxidant activity via the activation of the Nrf2-ARE pathway			[27]
Zedoarolide B (**85**)			Dual-luciferase reporter gene assay in 293 T cells	Activate the transcription of Nrf2 in 293 T cells			[32]
Alismoxide (**3**)		Anti-aging effect	UVB-mediated HaCaT cell model	Inhibit the production of MMP-1 in UV-irradiated HaCaT cells			[149]
Zedoarondiol (**18**)		Antifungal activity	Broad-spectrum antifungal activities	Exhibit broad-spectrum antifungal activities	*A. brassicicola*: 100 μg/mL; *P. parasitica var. nicotianae*: 100 μg/mL; *C. capsici*: 50 μg/mL; *B. oryzae*: 100 μg/mL; *D. medusaea Nitschke*: 100 μg/mL; *C. paradoxa Moreau*: 25 μg/mL; *E. turcicum*: 50 μg/mL; *P. theae*: 25 μg/mL; *A. citri*: 50 μg/mL	*A. brassicicola*: 12.5 μg/mL; *P. parasitica var. nicotianae*: 50 μg/mL; *C. capsici*: 12.5 μg/mL; *B. oryzae*: 50 μg/mL; *D. medusaea Nitschke*: 50 μg/mL; *C. paradoxa Moreau*: 25 μg/mL; *E. turcicum*: 12.5 μg/mL; *P. theae*: 25 μg/mL; *A. citri*: 25 μg/mL (Prochloraz)	[36]
		Endothelial cell injury protective effect	ox-LDL-induced HUVEC injury	Attenuate ox-LDL-induced endothelial cell injury by inhibiting oxidative stress and inflammation via the Nrf2/HO-1 pathway			[121]
Isozedoarondiol (**20**)		Antifungal activity	Broad-spectrum antifungal activities	Exhibit broad-spectrum antifungal activities	*A. brassicicola*: 100 μg/mL; *P. parasitica var. nicotianae*: 100 μg/mL; *C. capsici*: 100 μg/mL; *B. oryzae*: 50 μg/mL; *D. medusaea Nitschke*: 100 μg/mL; *C. paradoxa Moreau*: 25 μg/mL; *E. turcicum*: 50 μg/mL; *P. theae*: 50 μg/mL; *A. citri*: 50 μg/mL	*A. brassicicola*: 12.5 μg/mL; *P. parasitica var. nicotianae*: 50 μg/mL; *C. capsici*: 12.5 μg/mL; *B. oryzae*: 50 μg/mL; *D. medusaea Nitschke*: 50 μg/mL; *C. paradoxa Moreau*: 25 μg/mL; *E. turcicum*: 12.5 μg/mL; *P. theae*: 25 μg/mL; *A. citri*: 25 μg/mL (Prochloraz)	[36]
		Anti-aging effect	UVB-mediated HaCaT cell model	Inhibit production of MMP-1 in UV-irradiated HaCaT cells			[149]
Procurcumenol (**29**)		Antioxidant activity	Nrf2-luciferase activity in HEK 293 cells	Exhibit antioxidant activity via activation of the Nrf2-ARE pathway			[27]
		Antibacterial effect	Antibacterial activity against *S. albus*	Antibacterial activity against *S. albus*	*S. albus*: 1.25 μg/mL	*S. albus*: 0.6 μg/mL (Ciprofloxacin)	[40]
		Neuroprotective property	H_2_O_2_-induced oxidative stress in NG108-15 cells	Show moderate protection of NG108-15 cells	Neuroprotective: cell viability: 80.00 ± 0.71% (15 μM) (H_2_O_2_: 67.63 ± 0.86)		[143]
Isoprocurcumenol (**43**)		Skin function maintenance activity	UVB-induced cellular damage	Activate EGFR signaling, increase the phosphorylation of ERK and AKT, upregulate the expression of genes related to cell growth and proliferation, and induce the proliferation of keratinocytes			[142]
		Neuroprotective property; antioxidant activity	H_2_O_2_-induced oxidative stress in NG108-15 cells; oxygen radical antioxidant capacity assay	Show moderate protection of NG108-15 cells; antioxidant activity	Neuroprotective: cell viability: 80.96 ± 0.91% (4 μM) (H_2_O_2_: 67.63 ± 0.86) Antioxidant: TE: 26.43 ± 1.88 μM/100 μg		[143]
Curcumenol (**61**)		Neuroprotective property	H_2_O_2_-induced oxidative stress in NG108-15 cells	Show moderate protection of NG108-15 cells	Neuroprotective: cell viability: 103.04 ± 2.17% (4 μM) (H_2_O_2_: 67.63 ± 0.86)		[143]
		Improvement of intervertebral disc catabolism status	Lumbar spine-instability mouse model	Inhibit TNF*α*/NF-*κ*B signaling pathway and mitigate the expression of the MMP family (MMP-3, MMP-9, and MMP-13)			[146]
Germacrone (**116**)	Germacrane-type sesquiterpenoids	Neuroprotective property; antioxidant activity	H_2_O_2_-induced oxidative stress in NG108-15 cells; oxygen radical antioxidant capacity assay	Show moderate protection of NG108-15 cells; antioxidant activity	Neuroprotective: cell viability: 89.99 ± 2.01% (15 μM) (H_2_O_2_: 67.63 ± 0.86) Antioxidant: TE: 24.86 ± 2.33 μM/100 μg		[143]
		Anti-aging effect	UVB-induced damage in HaCaT cells	Inhibit UVB-induced upregulation of mRNA and protein expression levels of MMP-1, MMP-2, and MMP-3			[144]
Curdione (**117**)		Effect on sepsis-induced lung injury	CLP surgery established mice sepsis model	Inhibit platelet-mediated neutrophil recruitment, infiltration, and NET formation; exert anti-inflammatory and antioxidant properties			[145]
Dehydrocurdione (**121**)		Ca(2+) channel blocker-like effect	Ca(2+) channel blocker-like model	Exhibit a Ca(2+) channel blocker-like effect on rodent intestinal and vascular smooth muscles			[150]
		Neuroprotective property; antioxidant activity	H_2_O_2_-induced oxidative stress in NG108-15 cells; oxygen radical antioxidant capacity assay	Show obvious protection of NG108-15 cells; antioxidant activity	Neuroprotective: cell viability: 100.60 ± 1.72% (10 μM) (H_2_O_2_: 67.63 ± 0.86) Antioxidant: TE: 26.18 ± 2.59 μM/100 μg		[143]
Heyneanone D (**123**)		Antibacterial effect	Antibacterial activity against *E. coli*	Antibacterial activity against *E. coli*	*E. coli*: 1.25 μg/mL	*E. coli*: 0.3 μg/mL (Ciprofloxacin)	[40]
13-Hydroxygermacrone (**124**)		Anti-aging effect	UVB-induced damage in HaCaT cells	Inhibit UVB-induced upregulation of mRNA and protein expression levels of MMP-1, MMP-2, and MMP-3			[144]
Zederone (**143**)		Alzheimer’s disease	Aluminium-induced dementia rat model	Improve fecal microbiological profiles; regulate gut bacterial ecological imbalances			[147]
		Antioxidant activity	Oxygen radical antioxidant capacity assay	Antioxidant	TE: 27.78 ± 2.53 μM/100 μg		[143]
Curcolide (**202**)	Eudesmane-type sesquiterpenoids	Antioxidant property	Dual-luciferase reporter gene assay in 293 T cells	Activate the transcription of Nrf2 in 293 T cells			[32]
Curcumanolide A (**241**)	Other-type sesquiterpenoids	Relaxant effect on uterine smooth muscle	Oxytocin-induced contraction model of rat uterine smooth muscle	Show an inhibitory effect against oxytocin-induced rat uterine smooth muscle contraction			[15]
Curcumenone (**258**)		Protective effect on drunkenness	Alcohol-induced drunkenness model	Increase liver alcohol dehydrogenase activity and decrease the elevation of blood alcohol concentrations			[148]
		Antioxidant activity	Oxygen radical antioxidant capacity assay	Antioxidant	TE: 21.16 ± 2.12 μM/100 μg		[143]
